# Combined metabolomics and bioactivity assays kernelby-productsof two native Chinese cherry species: The sources of bioactive nutraceutical compounds

**DOI:** 10.1016/j.fochx.2024.101625

**Published:** 2024-07-05

**Authors:** Ziwei Wang, Lin Li, Jiaqi Han, Xinyu Bai, Binbin Wei, Ronghua Fan

**Affiliations:** a Department of Sanitary Inspection, School of Public Health, Shenyang Medical College, Shenyang 110034, China; b Developing Pediatric department of Shengjing Hospital, China Medical University,No.36Sanhao Street, Shenyang 110000, China; c School of Pharmacy, China Medical University, No.77 Puhe Road, Shenyang 110122, China

**Keywords:** Antioxidant, Antiproliferation, Untargeted metabolomics, Cherries, Food by-products

## Abstract

Cherry kernels are a by-product of cherries that are usually discarded, leading to waste and pollution. In this study, the chemical composition of 21 batches of cherry kernels from two different cherry species was analyzed using untargeted metabolomics. The in vitro antioxidant activity, cellular antioxidant activity, and antiproliferative activity of these kernel extracts were also determined, and a correlation analysis was conducted between differential compounds and biological activity. A total of 49 differential compounds were screened. The kernels of *Prunus tomentosa* were found to have significantly higher total phenol, total flavonoid content, and biological activity than those of *Prunus pseudocerasus* (*P* < 0.05). Correlation analysis showed that flavonoids had the greatest contribution to biological activity. The study suggests that both species of cherry kernel, particularly *Prunus tomentosa*, could be a potential source of bioactive compounds that could be used in the pharmaceutical, cosmetic, and food industries.

## Highlights

• The kernel metabolic profiles of the two species were clearly distinguished.
• 49 differential compounds were identified from both species kernels.
• The kernel of PT showed significantly higher bioactivity than that of PP.
• Correlation analysis revealed 12 compounds as key bioactive compounds.

## 1 Introduction

Managing food waste properly has become increasingly important. The most preferred methods for managing food waste are preventing its generation and reusing it. However, it is not always possible to completely prevent food waste generation because significant amounts of residues and byproducts are produced during food processing(Lee et al., 2022). Globally, the by-products of plant food processing have garnered significant attention from both the industry and the scientific community. This is because such by-products have been shown to contain valuable compounds in large quantities that can be recovered and used as natural food ingredients or biologically active components. Secondary metabolites of plants, including phenolic compounds and carotenoids, as well as high-molecular-mass constituents of the plant cell wall, have been the subject of extensive research. These secondary metabolites are a critical component of a plant's defense system, and they exhibit a range of activities, such as antimicrobial, antiproliferative, and antioxidant activities(Schieber, 2017). Therefore, utilizing food waste would be a preferred method of managing food waste.

There are four main widely grown cherry species in China, of which *Prunuspseudocerasus* (PP) and *Prunus tomentosa* (PT) are native to China(Cao et al., 2015). The PP, known as a fruit crop *gem*, has grown in prominence in China's cherry industry because to its exceptional nutrition, appealing colors, and delectable tastes(Z. Liu et al., 2024). The fruits of PT have many significant characteristics, such as their distinctflavors, diversecolors, and high vitamin and antioxidant content(Zhang et al., 2022). While PP and PT cherry fruits are edible when raw, they work best when processed to make jams, preserves, fruit juices, and health-promoting extracts. During consumption and processing, kernels, pits, and stones are the main by-products.

Frequently, stones, pits, and fruit kernels are considered unwanted components of fruits. However, they have significant industrial potential and can be quite valuable. Besides their use in the food industry, particularly for the production of edible oils, seeds have also been utilized for medicinal and cosmetic purposes in recent times(Senica et al., 2016). Fruit kernels are rich sources of oils that contain essential fatty acids, tocochromanols, carotenoids, phytosterols, and squalene, which are functional bioactive compounds (Górnaś et al., 2016). In previous studies, researchers have found a number of compounds, such as phenylpropanoid sucrose esters, phenylpropanoids, lignans, dihydrobenzofuranneolignans, flavonoids, etc. in PT kernels. These compounds have biological activities, such as antiproliferative, β-amyloid aggregation inhibition, antioxidant activity, and the inhibitory activity of nitric oxide production(Kim et al., 2008; Q. Liu et al., 2019; Q. B. Liu et al., 2014; Zhao et al., 2014). On the other hand, the chemical composition or biological activity of PP kernel has not been reportedandthere is a lack of sufficient research to conduct systematic comparative studies on the composition and biological activity of the two species.

For these reasons, in this study, the metabolic profile of the samples was evaluated by ultra-performance liquid chromatography with quadrupole time-of-flight mass spectrometry (UPLC-QTOF/MS) and performed a metabolomic workflow on the 21 batches of kernel of two cherry species, to establish the chemical profile of these samples, enabling the determination of differential compounds. The present study also evaluated the in vitro antioxidant activity, cellular antioxidant activity, antiproliferative activity, cytotoxicity, cell cycle and apoptosis analysis of these sample extracts. In addition, chemometric tools were used to establish the relationship between differential compounds and biological activity, enabling the screening for key bioactive compounds. In this way, it was possible to assess the value of these food by-products generated in the fruit agroindustry.

## 2 Materials and methods

### 2.1 Chemicals and reagents

Aladdin Industrial Corporation (Shanghai, China) provided the following: 2′,7’-Dichlorodihydrofluorescein diacetate (DCFH-DA), 2,2′-Azino-bis(3-ethylbenzthiazoline-6-sulfonic acid) (ABTS), 6-hydroxy-2,5,7,8-hydroxy-2,5,7,8-tetramethyl chroman-2-carboxylic acid (Trolox), and 2,2′-Azobis(2-methylpropionamidine) dihydrochloride (AAPH).The supplier of 2,2-diphenyl-1-picrylhydrazyl (DPPH) was Macklin Biochemical Co. Ltd. located in Shanghai, China.Yuanye Biotechnology Ltd. (Shanghai, China) provided thekaempferol, caffeic acid,rutin,ascorbic acid,paeonol, vanillic acid, apigenin,chlorogenic acid, naringin, ferulic acid, hyperoside, liquiritin, quercetin,and folin-Ciocalteu reagent. Annexin V-FITC cell apoptosis assay kit and cell cycle assay kit were purchased from Beyotime (Beijing, China).The purity of all other chemicals meets the requirements of the respective experiments.

### 2.2 Plant materials

**Fig. 1:**
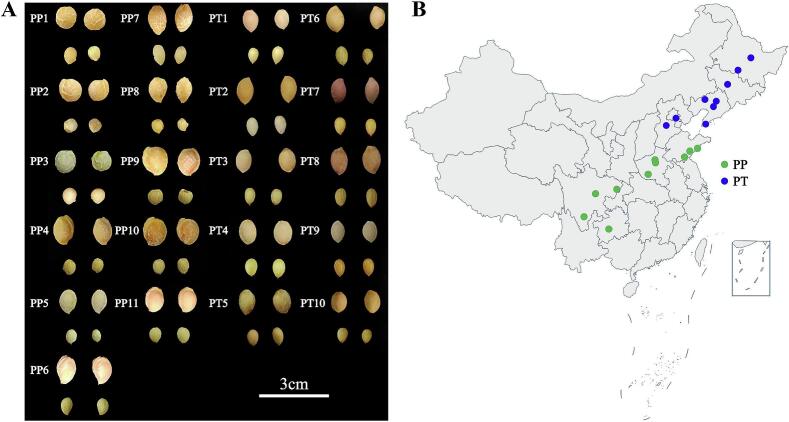
Photographs of 21 batches of samples (A). Sampling point for 21 batches of samples (B). PP, *Prunus pseudocerasus*; PT, *Prunus tomentosa*.

**Fig. 2:**
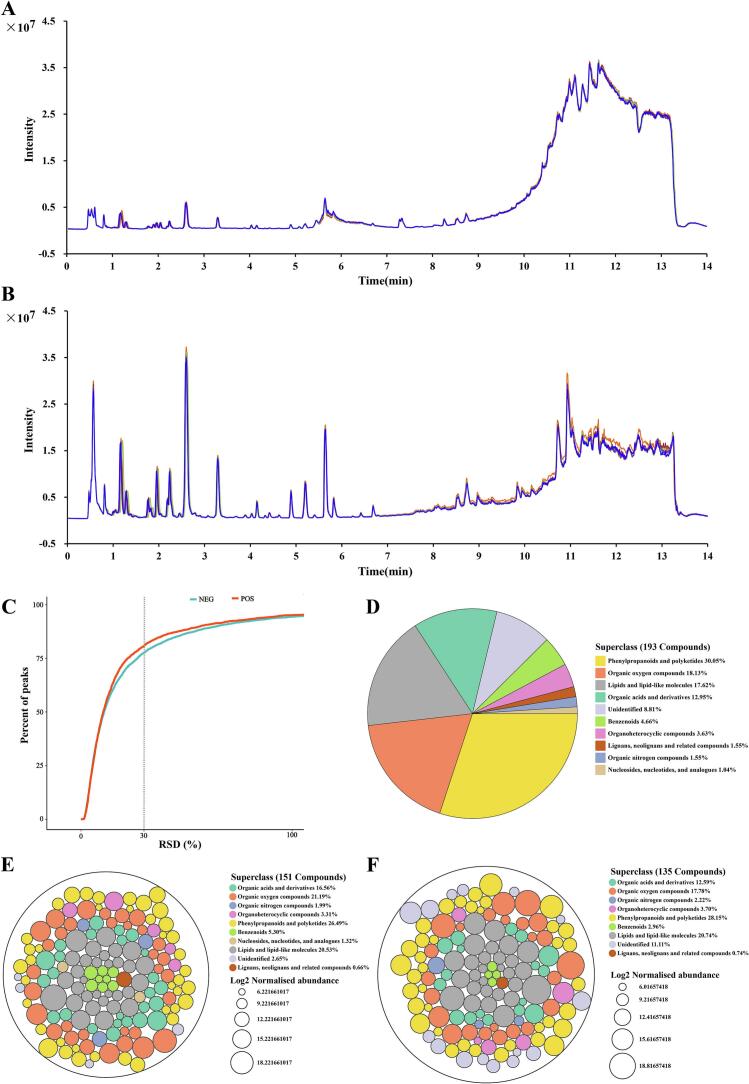
Total ion chromatogram (TIC) of five QC sample from positive (A) and negative (B) ESI modes.Relative standard deviation (RSD%) of QC samples under positive ion mode and negative ion mode (C). Biochemical classification and proportion of all identified compounds (D). Distribution of compounds in different species, each circle represents a compound, the color represents the classification of the compound and the size represents the average Log_2_ Normalized abundance of that compound. Compounds identified in PP (E). Compounds identified in PP (F).

**Fig. 3:**
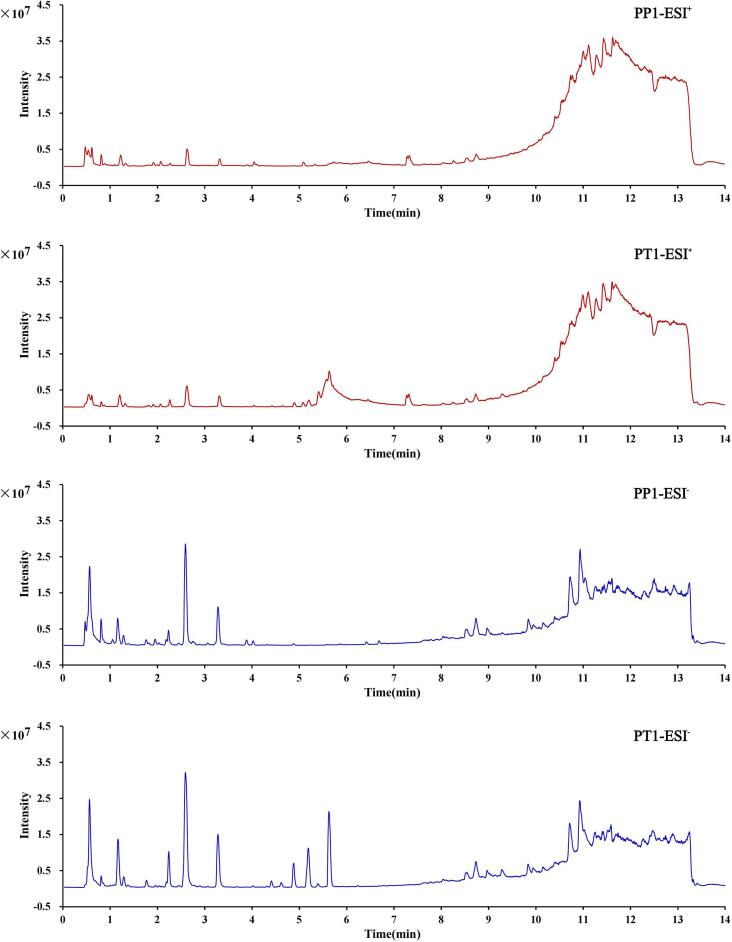
The representative total ion chromatogram (TIC) of Prunus pseudocerasus and *Prunus tomentosa* was acquired in positive (ESI^+^) and negative (ESI^−^) ionization modes.

PP and PT were harvested from different locations or different varieties from the same locations(Fig. 1Aand B). Samples were collected between April and July 2022 (Table 1). Over 50 fruits, free from decay and mechanical damage, were selected for each batch. The pulp was removed, and kernels were taken out. The kernels were then freeze-dried, ground into powder, and sieved through a 60 mesh sieve. After that, they were placed inside opaque centrifuge tubes and kept cold, at −80 °C, until needed.Stored plant samples were extracted for experiments within a year. (See Fig. 1, Fig. 2, Fig. 3.)

**Table 1:** Details of 21 batches of samples.

**Samples**	**Source**	**Edible rate (%)**	**Date of collection**
*P. pseudocerasus*			
PP1	Chengdu City, Sichuan Province.	89.63%	2022.04.06
PP2	Anshun City, Guizhou Province	87.63%	2022.04.13
PP3	Liangshan Yi Autonomous Prefecture, Sichuan Province	89.88%	2022.04.21
PP4	Nanyang City, Henan Province	89.41%	2022.04.21
PP5	Dazhou City, Sichuan Province	90.41%	2022.04.25
PP6	Zhengzhou City, Henan Province	88.58%	2022.05.02
PP7	Linyi City, Shandong Province	86.68%	2022.05.03
PP8	Xinzheng City, Henan Province	88.96%	2022.05.06
PP9	Qingdao City, Shandong Province	81.57%	2022.05.06
PP10	Rizhao City, Shandong Province	86.45%	2022.05.13
PP11	Rizhao City, Shandong Province	83.83%	2022.05.13

*P.tomentosa*			
PT1	Baoding City, Hebei Province	82.76%	2022.06.06
PT2	Langfang City, Hebei Province	80.66%	2022.06.07
PT3	Shenyang City, Liaoning Province	84.69%	2022.06.08
PT4	Dalian City, Liaoning Province	82.76%	2022.06.10
PT5	Anshan City, Liaoning Province	91.67%	2022.06.12
PT6	Fuxin City, Liaoning Province	86.16%	2022.06.22
PT7	Changchun City, Jilin Province	85.72%	2022.06.23
PT8	Shenyang City, Liaoning Province	90.98%	2022.06.25
PT9	Yichun City, Heilongjiang Province	88.52%	2022.06.27
PT10	Harbin City, Heilongjiang Province	85.68%	2022.07.06

### 2.3 Sample extraction

The sample extraction was carried out using the previously reported method(Z. Wang et al., 2023). To briefly summarize, 160 mg of each sample was mixed with 3.2 mL of mixed solvent (methanol: water at a 2:3 ratio) in a 10 mL centrifuge tube. The mixture was then sonicated for 10 min at room temperature with the help of an ultrasonic bath (power 500 W). The samples were centrifuged at 10000 ×*g* at 4 °C for 10 min and the supernatant was collected and preserved. This process was repeated three times, and the supernatants were combined. The combined supernatants were then diluted with mixed solventto a final volume of 10 mL. The extraction process was performed three times for each sample. Andthe extracts were brought to 4 °C overnight to precipitate the proteins and then filtered through a 0.22 μm membrane. Until additional analysis, the extracts were kept at −20 °C in storage.The storage time of the extract is not more than three days.

### 2.4 Determination of total phenolcontent (TPC) andtotal flavonoid content (TFC),and in vitroantioxidant activity

TPC, TFC, and in vitro antioxidant activities (DPPH radical scavenging capacity, ABTS radical scavenging capacity, and FRAP) were determined according to the previous methods(Z. Wang et al., 2023). The detailed experimental method is as follows.

The determination of TPC using Folin-Ciocalteu reagent, gallic acid was used as a standard. 200 μL of appropriately diluted samples were mixed with 1.5 mL of Folin-Ciocalteu reagent (diluted 1:5 in distilled water), vortexed and homogenized for 30 s, and incubated for 2 min at room temperature. Then, added 1.2 mL of sodium carbonate solution (7.5%, *w*/*v*) and incubated the samples for 60 min at room temperature in the dark. The absorbance of the samples and calibration curve standards were measured at 765 nm. A blank consisting of water and samples was used as a reference. Sample color blanks were checked using samples and water to exclude color interference from sample extracts. Data were calculated by comparing the standard curve (10–100 μg/mL gallic acid) with the absorbance of each sample. The results were expressed in milligram equivalent of gallic acid (GAE) per gram of sample dry weight (mg GAE/ g dw).

TFC was determined by the spectrometric method, using rutin as a standard. In short, 2.7 mL of appropriately diluted extract was added to 150 μL of NaNO_2_ solution (5% *w*/*v*) and mixed. After 5 min, 150 μL of AlNO_3_ (10% w/v) was added. After 6 min, 2 mL of 1.0 mol/L NaOH was added. The absorbance at 515 nm was measured by incubating for 10 min at room temperature in the dark. Data were calculated by comparing the standard curve (5–100 μg/mL rutin) with the absorbance of each sample. The results were expressed as milligram equivalents of rutin (RE) per gram of sample dry weight (mg RE/ g dw).

The antioxidant activity of the samples was assessed by DPPH radical scavenging activity assay with appropriate modifications. Briefly, 0.5 mL of appropriately diluted sample extract was mixed with 2.5 mL of freshly prepared DPPH radical methanol solution (80 μmol/L). After standing in the dark for 2 h, the absorbance was measured at 515 nm. Trolox was used as a standard for creating calibration curves (20–200 μmol/L). The results of antioxidant activity were expressed as μmol of Trolox (TE) equivalent antioxidant capacity per gram of the sample dry weight (μmol TE/g dw).

The ABTS radical scavenging activity assays were performed using a previous method with minor modifications. ABTS working solution was prepared by mixing potassium persulfate (2.45 mmol/L) and ABTS (7 mmol/L) (1:1, *v*/v) and incubated at room temperature and protected from light for 12–16 h. The working solution was then diluted with distilled water to obtain an absorbance value of 0.70 ± 0.02 at 734 nm. Next, 200 μL of the appropriately diluted sample solution was mixed with 2.8 mL of ABTS working solution. The mixture was then incubated in the dark at 25 °C for 6 min and its absorbance at 734 nm was measured. Trolox was used as a standard for creating the calibration curve (30–300 μmol/L). The results of antioxidant activity were expressed as μmol of Trolox equivalent antioxidant capacity per gram of the sample dry weight (μmol TE/g dw).

The ferric-reducing antioxidant capacity (FRAP) assay was based on a previous method with some modifications. The FRAP solution consisted of 2.5 mL 10 mmol/L TPTZ solution (0.31 g TPTZ dissolved in 100 mL of 40 mmol/L HCl), 2.5 mL FeCl_3_·6H_2_O water solution (20 mmol/L) and 25 mL acetate buffer (0.3 mol/L, pH = 3.6). The mixture was heated to 37 °C before use. 200 μL of the appropriately diluted sample solution was mixed with 2.8 mL of FRAP working solution for 30 min at room temperature and its absorbance was measured at 593 nm. Trolox was used as a standard for creating the calibration curve (40–400 μmol/L). The results of antioxidant activity were expressed as μmol of Trolox equivalent antioxidant capacity per gram of the sample dry weight (μmol TE/g dw).

Since TPC, TFC, DPPH, ABTS, and FRAP are all traditional abiotic Antioxidant activities assays, they were combined as antioxidant potency index (ACI) in the subsequent analyses as previously reported(Z. Wang et al., 2023).

### 2.5 UPLC-ESI-QTOF/MS^E^-based untargeted metabolomics analysis

To monitor the stability of the instrumental system, quality control (QC) samples were prepared by mixing aliquots of all samples measured, andrepeat analysis of QC samples after every three test samples.

The UPLC system consisted of an ACQUITY UPLC instrument system (Waters, Milford, MA, USA), an ACQUITY UPLCTM T3 column (Walsch, Milford, USA), and the whole system was controlled by MassLynx4.1 software. The mobile phase system for UPLC consisted of water containing 0.1% formic acid (solvent A) and acetonitrile containing 0.1% formic acid (solvent B), and the separation was carried out according to the following optimized elution program: 0–4 min, 90%–70% A; 4–10 min, 70%–0% A; 10–12 min, 0% A; 12–12.1 min, 0%–90% A;and 12.1–14 min, 90% A. The flow rate was 0.4 mL/min, the column temperature was 30 °C, the injection volume was 2 uL, and each sample was injected and detected in both positive and negative ion modes.

The mass spectrometry data were obtained by a Xevo G2QTOF (Waters, Milford, MA, USA) mass spectrometer equipped with a *Z*-SprayESI ion source. Mass spectral information was acquired using both normal and negative ion modes. Positive ion mode capillary voltage was 3.0 kV, negative ion mode capillary voltage was 2.5 kV, and sampling cone voltage was 30 V. The desolvation temperature and source temperature were set to 500 °C and 120 °C, respectively; nitrogen was used as the carrier gas in the mass spectrometry system, and the desolvation gas flow was set to 800 L/h and the cone gas flow was set to 50 L/h. The mass spectrometry data were collected in Continuum MS^E^mode, and two scanning functions were used, namely, low-energy (Function 1) and high-energy (Function 2), and the scanning mass range was 50–1200 Da. In the low-energy function, the particle collision energy was 6 V, and the scan time was 0.2 s. Under the high-energy scan function, the particle collision energy was repeated back and forth between the range of 20–40 V, and the scan time was also 0.2 s. Leucine-enkephalin (200 pg/μL) solution was used as lock-mass at a flow rate of 10 μL/min, producing reference fragment masses of *m*/*z* 556.2771 (positive ion mode) and m/z 554.2615 (negative ion mode), respectively.

Raw data collected by Masslynx4.1 software in all positive and negative ion modes were imported into Progenesis QI2.3 software (Waters, Milford, MA, USA). The mass spectrometry data is then subjected to data processing steps such as peak alignment, peak picking, normalization, and deconvolution. The default value for undetected peaks was 0. The data were filtered to remove unstable signals with RSD% > 30% in the QC samples. Peak picking retention time was limited to 0.3–12 min.

Preprocessed data were used for compound identification using Progenesis QI 2.3 software (Waters, Milford, MA, USA). Untarget identification was performed using a previously established Prunus genus database of 438 compounds and public databases (Phenol Explorer database, and HMDB). The parameters were set as follows: precursor tolerance>10 ppm, fragment tolerance>10 ppm, identification score generated by the software>35, and isotope similarity>85. The targeted identification of common plant compounds was also performed on kernels based on retention time, mass number, and MS-MS spectra of the comparison standards.

### 2.6 Cellular antioxidant activity (CAA), antiproliferative activity, cytotoxicity, cell cycle and apoptosisanalysis based on human colorectal adenocarcinoma (Caco-2) cells

#### 2.6.1 Cell culture

Caco-2 cells were cultured in MEM medium supplemented with 20% heat-inactivated fetal bovine serum and 1% penicillin-streptomycin. Cells were placed in a cell culture incubator at 37 °C with 5% CO_2_, and when cell confluence reached ∼80%, the cells were used for the next experiment.All experiments were done independently in triplicate per experimental point.

#### 2.6.2 CAA assay

All sample extracts were freeze-dried to remove water after blowing off the organic solvent with nitrogen, reconstituted with culture medium (all samples at a concentration of 20 mg/mL), and filtered through a 0.22 μm membrane. Quercetin standards were solubilized in DMSO and diluted to the appropriate concentration with culture medium for CAA experiments. The organic solvent content of all solutions acting on cells was <1%.

CAA was determined using a previously reported method(Wan et al., 2015a; Wolfe & Rui, 2007)with the following procedure: 100 μL of Caco-2 cells suspension at a concentration of 6 × 10^5^ cells/mL was added to a 96-well bottom-transparent black-well enzyme labeling plate, incubated for 24 h, and rinsed once with PBS. To each well, 50 μL of DCFH-DA (concentration 50 μmol/L) and 50 μL of sample or quercetin (QE) standard solution were added, and the plate was incubated in an incubator for 20 min, the medium was discarded, and the plate was rinsed twice with PBS. Then 100 μL of AAPH solution (600 μmol/L) was added. Control wells were treated with DCFH-DA without antioxidants and AAPH, and blank wells were treated with DCFH-DA without AAPH and antioxidants. Subsequently, the plates were placed in a multifunctional enzyme marker to monitor the changes in fluorescence signals over a period of 60 min, with the excitation wavelength of 485 nm and the emission wavelength of 538 nm measured every 5 min. The effect of the antioxidant treatments on the Caco-2 cell line was quantified by detecting the percentage of fluorescence reduction. A total of 13 fluorescence response readings were generated to produce a curve line, and the percentage reduction in the area under the curve was calculated to measure the antioxidant capacity of the extract.A quercetin standard curve was established with quercetin concentration (1–15 μmol/L) in the horizontal coordinate and CAA_unit_ in the vertical coordinate. The final result was expressed as quercetin (QE) equivalents per 100 g dry weight (μmol QE/100 g dw).The percent reduction (or the CAA unit) was calculated as follows:

$${\mathrm{CAA}}_{\text{unit}}=\%\text{reduction}=\left(1-{\mathrm{AUC}}_{\text{sample}}/{\mathrm{AUC}}_{\text{control}}\right)*100$$

#### 2.6.3 Cytotoxicity and antiproliferative activity assays

Cellular antiproliferative activity and cytotoxicity assays were performed according to the previous test method(H. Wang et al., 2016), but the cell counting method was changed.Specifically, for cytotoxicity assays, cells were inoculated in 96-well plates at a density of 4 × 10^4^ cells/well. After 24 h of incubation in a 37 °C incubator, the medium was discarded and the cells were washed using PBS. Subsequently, the samples were diluted to a suitable concentration (10–60 mg/mL) using the complete medium, which was added to the well plates, and the cells were continued to be incubated for 24 h. After 24 h of incubation, the medium was discarded from the plates and the cells were washed using PBS. Then 100 μL of 10% CCK-8 was added to each well, and after incubation for 2 h, the absorbance of the reaction solution at 450 nm in each well was measured by an enzyme meter. Cytotoxicity was determined by CC_20_ (cytotoxic concentration at 20% cell death) and recorded as milligrams per milliliter (mg/mL).

For antiproliferative activity analysis, cells were inoculated in 96-well plates at a density of 2.5 × 10^4^ cells/well, incubated in a constant temperature incubator at 37 °C for 6 h. The medium was discarded, and the cells were incubated in a constant temperature incubator at 37 °C with the addition of a medium or a well-diluted sample of the medium (10–60 mg/mL) for 72 h. The medium was discarded, and the subsequent operations were the same as those in the cytotoxicity assay. The results were expressed as half inhibitory concentration (IC_50_) values and recorded as milligrams per milliliter (mg/mL).

### 2.7 Cell cycle analysis and apoptosis analysis

Based on the results of the antiproliferative activity experiment, appropriate concentrations (10,20,30 mg/mL) of PP (PP8) and PT(PT4) samples with the strongest antiproliferative activity were selected for cell cycle analysis or apoptosis detection. Inoculate Caco-2 cells into a 6-well plate with 5 × 10^5^ cells per well and incubate for 24 h. The cells were then harvested after incubation in a complete medium with different concentrations of PP extract or PT extract for 72 h and further treated for cell cycle analysis or apoptosis detection.

For cell cycle analysis, the cells were immobilized in 70% ethanol at 4 °C for 2 h. Subsequently centrifuged and washed with PBS, the cells were stained with 50 g/mL PI and measured by flow cytometry according to the manufacturer's protocol. The cell cycle distribution was then calculated using ModFit LTTM software (Becton Dickinson, USA).

Apoptosis was detected by flow cytometry according to the manufacturer's instructions. Then FlowJo software (Tree Star, V.10.0.7, USA) was used. The cells were divided into living cells, dead cells, and apoptotic cells, and then the percentage of apoptotic cells in each group was compared.

### 2.8 Statistical analysis

Experiments were conducted in triplicate and expressed as mean ± standard deviation. One-way analysis of variance (ANOVA) test and Duncan's post hoc test were used to assess statistical differences between samples using SPSS 19.0 software (SPSS Inc., Chicago, Illinois, USA). And one-tailed or two-tailed Student's *t*-test was used for two-group comparisons. *P* < 0.05 represents a significant difference. Pearson's correlation coefficient as well as the area under the CAA curve were calculated by GraphPad data analysis software (Version Prism 8.0). Untargeted metabolomics data were subjected to principal component analysis (PCA) and orthogonal partial least squares discriminant analysis (OPLS-DA) using SIMCA 14.1 (Umetrics, Umeå, Sweden) using Pareto scaling mode.

## 3 Results and discussion

### 3.1 Compounds identifications

**Fig. 4:**
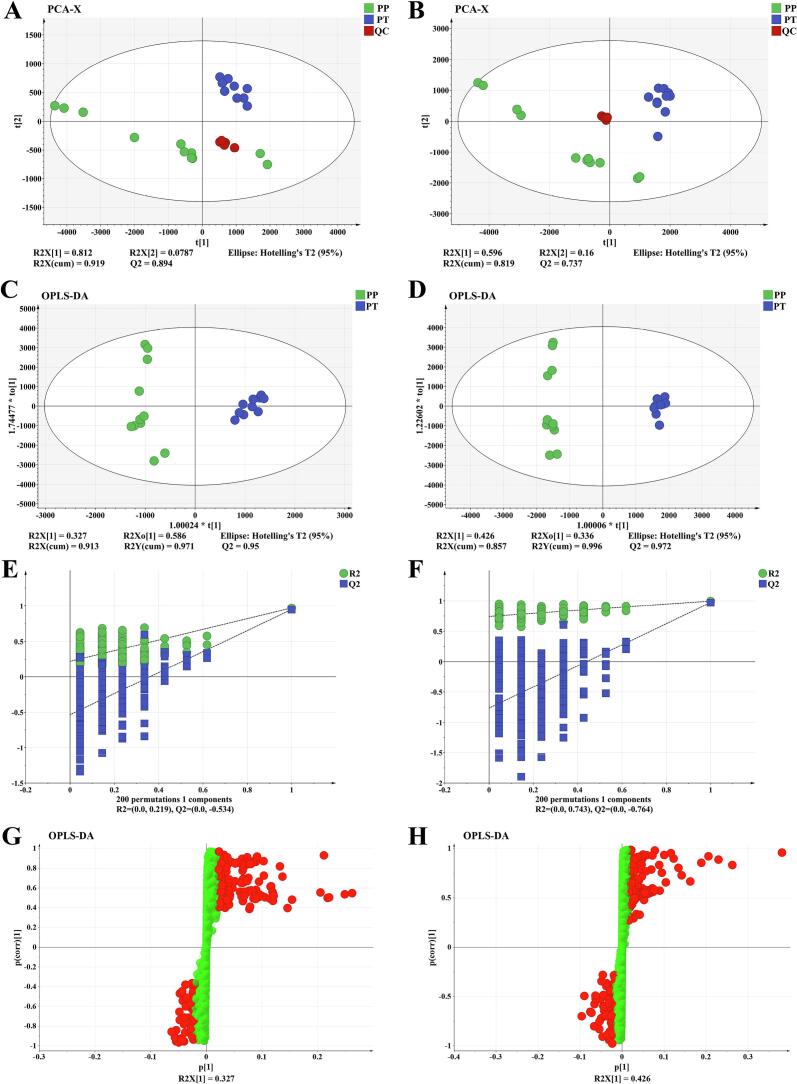
Positive and negative ion mode principal component analysis model plot (A and B). Positive and negative ion mode Orthogonal partial least-squares discriminant analysis (OPLS-DA) model plots (C and D). OPLS-DA 200 X permutation tests in positive and negative ion modes (E and F). S-plot, red dots represent features with VIP >1. Positive ion mode(G). Negative ion mode(H).PP, *Prunus pseudocerasus*; PT, *Prunus tomentosa*. (For interpretation of the references to color in this figure legend, the reader is referred to the web version of this article.)

Overlay analysis of the total ion current (TIC) plots of the five positive-ion mode or negative-ion mode quality control (QC) samples shows that the TIC plots are highly overlapping in both positive-ion mode and negative-ion mode QC samples(Fig. 2 A and B). This indicates that the same samples measured at different times maintained a high degree of consistency. By calculating the relative standard deviation (RSD%) value of each characteristic peak among the QC samples, >70% of the peaks in the positive-negative ion mode QC samples had RSD% values less than or equal to 30% (Fig. 2C), which proves that the method has good stability and repeatability, and this data is reliable.In addition, the QC samples were closely clustered in the PCA plots (Fig. 4A and B), which also indicates that the test results are reliable.

A total of 193 compounds were identified from 21 batches of samples by comparing self-constructed databases, public databases, and standards (Supplementary data 1). According to the classification information of the compounds in the HMDB database, the identified compounds were categorized into 10 superclasses, which included 58 phenylpropanoids and polyketides, 35 organic oxygen compounds, 34 lipids, and lipid-like molecules, 25 organic acids and derivatives, 9 benzenoids, 7 organoheterocyclic compounds, 3 lignans, neolignans, related compounds, 3 Organic nitrogencompounds, 2 nucleosides, nucleotides, and analogues, and 17 undefined compounds(Fig. 2D). They contained sixteen pairs of isomers that could not be distinguished,and 15 isomers with different retention times than the standard but the same chemical formula.

The TIC plots of the kernel extracts of the two species show that there is a difference in the chemical composition and in the metabolite levels (Fig. 3). Circle packing plots (Fig. 2EandF) show compounds identified by kernels of both species. In this study, compounds present in 80% of the batch samples of each species were considered to be compounds contained by that species, 151 and 135 compounds were identified in PP and PT, respectively. Both species have only 102 compounds in common, and there are huge differences in the type and content of compounds. To more clearly illustrate the identification process of compounds in this study, we take the identification process of compounds Quercitrin, rutin, and rutin isomer as examples (Supplementary Fig.S1-S2).

### 3.2 Differential compounds analysis based on PCA and OPLS-DA

Principal Component Analysis (PCA) is the most commonly used dimensionality reduction method and has a wide range of applications in data compression and redundancy elimination (Nguyen et al., 2023). The pre-processed data were analyzed by PCA in positive and negative ion mode, respectively.In this analysis, the positive ion mode PC1 explained 81.21% of the variance in the data and PC2 explained 7.87% of the variance (Fig. 4A). Whereas negative ion mode PC1 and PC2 explained 59.64 and 16.04% of the total variance respectively (Fig. 4B). The samples of the same species were tightly coupled together. These findings suggest a clear trend of separation between PP and PT due to differences in metabolites.

The OPLS-DA analysis used partial least squares regression to model the relationship between metabolite expression and sample category to achieve the prediction of sample category, and the result was shown in Fig. 4C and D. The parameters of the OPLS-DA evaluation model are R2X, R2Y and Q2, where R2X and R2Y indicate the explanation rate of the X and Y matrices of the constructed model, respectively, and Q2 denotes the predictive ability of the model, and the closer the three indexes are to 1 indicates that the model is more stable and reliable, and a model can be regarded as a valid model when Q2 > 0.5, and an excellent model when Q2 > 0.9(Maritha et al., 2022). These parameters in this study were found in the positive ion mode (R2X = 0.913, R2Y = 0.971, Q2 = 0.950) and in the negative ion mode (R2X = 0.857, R2Y = 996, Q2 = 0.972), suggesting that the model performs excellently in both the positive and negative ion modes.In the OPLS-DA plot, PP and PT are distributed on both sides of the Y-axis, demonstrating that the kernels of the two species differ significantly in their metabolic profiles.In order to prevent the model from overfitting, the model was subjected to 200 permutation tests, and the final results are shown in Fig. 4E and F. The Q2 values of the stochastic model are smaller than those of the original model, indicating that the original model has good soundness of fit and does not have overfitting phenomenon.Based on these results OPLS-DA modeling can be used for further screening of differential compounds.

To screen for differentially expressed compounds between kernels of the two species, importance for projection (VIP) values were calculated using the OPLS-DA model in SIMCA-P software. Compounds with VIP ≥1 are usually selected as differential compounds. In the S-plot, feature points with VIP greater than one are marked in red (Fig. 4G and H). Differential compounds were then further selected by combining FC ≥ 2 or ≤ 0.5 and *P* < 0.05.

**Fig. 5:**
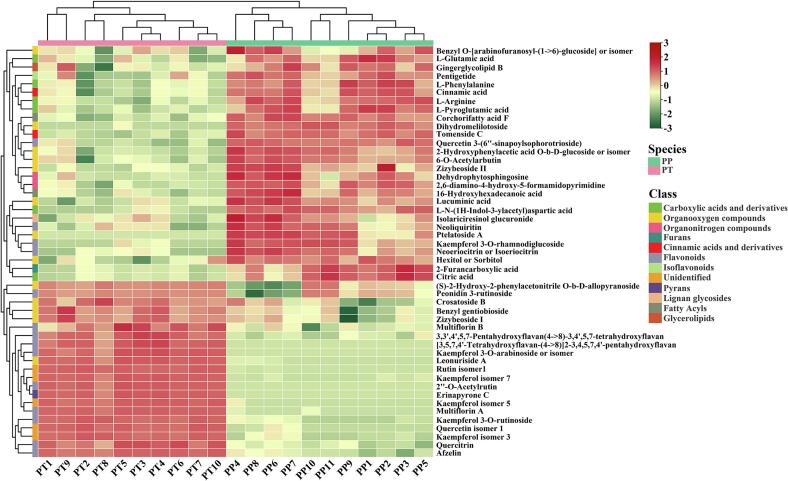
Hierarchical cluster analysis and heat map of 49 differential compounds in two species of kernels. Red to green color represents the relative abundance of compounds from high to low.PP, *Prunus pseudocerasus*; PT, *Prunus tomentosa*. (For interpretation of the references to color in this figure legend, the reader is referred to the web version of this article.)

The heatmap clearly shows the differential compounds between different kernels of two species, among which the number of differential compounds was 49 (28 compounds are higher in PP, and 21 compounds are higher in PT) between PP and PT. Out of all the compounds, 14 flavonoids accounted for 28.57% and 13 organooxygen compounds accounted for 26.54%. The remaining compounds included 6 carboxylic acids and derivatives, 5 unidentified compounds, 2 organonitrogen compounds, 2 cinnamic acids and derivatives, 2 fatty acyls, 1 furans, 1 isoflavonoids, 1 pyrans, 1 lignan glycosides, and 1 glycerolipids.Fig. 5 demonstrates the distribution and relative content of these differential compounds, and based on the results of the cluster analysis, the two species can be classified into two groups, with large differences in the composition of the two species.

### 3.3 TPC and TFC

Phenolic compounds are present as secondary metabolites in various types of plants. These plants, because they grow in wild environments, produce large amounts of phenolic compounds to resist environmental impacts and threats from other organisms.Phenolic compounds are a large group of secondary metabolites, including analytically simple compounds such as phenolic acids, as well as structurally complex compounds such as flavonoids(de Araújo et al., 2021). TPC and TFC, although named as content determinations, are also one of the commonly used indicators to reflect the antioxidant capacity of extracts(Rahim et al., 2021). As shown in Table 2, the average value of PT exhibited a generally higher TPC over those of PP (*P* < 0.01). The three batches of samples with the highest TPC are all PT. The TPC of both species samples was around 1.59–4.74 mg GAE/g dw. According to previous reports, a TPC over 0.5 mg GAE/ g was classified as a high category(Rufino et al., 2010; Vasco et al., 2008).All samples in this experiment fall into the high TPC category.

**Table 2:** Total phenoliccontent (TPC),Total flavonoid content (TFC), in vitro antioxidants,antioxidant potency index (ACI) cellular antioxidant activity (CAA), antiproliferative activity, and cytotoxicity of 21 batches of kernels (mean ± SD, n = 3), values with no letters in common in each column are significantly different (*P* < 0.05).

**Samples**	**TPC**(mg GAE/g dw)	**TFC (**mg RE/g dw**)**	**DPPH**(μmol TE/g dw)	**ABTS** (μmol TE/g dw)	**FRAP** (μmol TE/g dw)	**ACI**	**CAA**(μmol QE/100 g dw)	**Antiproliferative Activity** IC_50_ (mg/mLdw)	**Cytotoxicity** CC_20_ (mg/mLdw)
*P. pseudocerasus*									
PP1	2.15 ± 0.02 l	1.02 ± 0.04 l	6.09 ± 0.27 k	7.70 ± 0.21j	5.77 ± 0.18hi	37.14	8.82 ± 1.04 m	59.59 ± 1.70a	>60
PP2	3.09 ± 0.00 g	1.44 ± 0.10ij	10.54 ± 0.25d	11.62 ± 0.34de	9.18 ± 0.09c	57.35	30.30 ± 4.95efgh	51.23 ± 1.55bc	58.60 ± 1.43
PP3	2.48 ± 0.07 k	1.19 ± 0.05 k	5.58 ± 0.10 l	8.57 ± 0.08 h	5.51 ± 0.10i	39.09	15.32 ± 0.81klm	38.96 ± 0.05e	54.04 ± 3.84
PP4	3.41 ± 0.03e	1.16 ± 0.05 kl	6.05 ± 0.10 k	8.57 ± 0.12 h	5.88 ± 0.11 h	44.03	25.83 ± 1.33fghij	35.05 ± 0.76f	40.79 ± 2.43
PP5	2.50 ± 0.01jk	1.83 ± 0.12 g	9.98 ± 0.11e	12.01 ± 0.16de	9.17 ± 0.24c	56.33	11.19 ± 1.35 lm	47.90 ± 1.67 cd	47.23 ± 0.55
PP6	3.89 ± 0.07c	1.37 ± 0.05i	8.90 ± 0.08f	12.05 ± 0.07d	7.55 ± 0.14f	56.29	31.36 ± 8.31defg	35.00 ± 0.74f	46.17 ± 2.23
PP7	3.66 ± 0.09d	1.47 ± 0.06ij	8.34 ± 0.18 g	12.58 ± 0.31c	7.87 ± 0.21e	56.02	48.66 ± 1.17b	32.76 ± 1.03 fg	47.48 ± 7.45
PP8	2.94 ± 0.05 h	1.03 ± 0.06 l	4.70 ± 0.09 m	9.91 ± 0.08 g	4.95 ± 0.08j	39.53	27.52 ± 5.47efghi	32.69 ± 1.55 fg	50.14 ± 1.81
PP9	2.59 ± 0.10j	1.66 ± 0.17 h	5.89 ± 0.24kl	8.09 ± 0.14i	5.83 ± 0.31hi	42.25	18.53 ± 1.48jkl	46.32 ± 3.04d	>60
PP10	1.67 ± 0.09n	1.06 ± 0.03kl	4.29 ± 0.09n	6.55 ± 0.33j	4.41 ± 0.09 k	29.92	14.94 ± 1.98klm	49.86 ± 1.65bcd	>60
PP11	1.59 ± 0.04n	0.83 ± 0.06 m	4.41 ± 0.27mn	6.38 ± 0.38j	3.89 ± 0.28 l	27.76	23.99 ± 5.71ghij	49.18 ± 2.72bcd	>60
*P.tomentosa*									
PT1	2.77 ± 0.08i	1.55 ± 0.08 hi	5.68 ± 0.11 l	10.08 ± 0.09 fg	5.82 ± 0.10hi	43.99	20.7 ± 3.85ijk	51.94 ± 1.71b	>60
PT2	3.39 ± 0.07ef	2.07 ± 0.06f	7.13 ± 0.22i	11.59 ± 0.18e	7.73 ± 0.24ef	55.07	40.99 ± 8.03c	22.18 ± 2.26j	>60
PT3	4.69 ± 0.05ab	4.14 ± 0.14a	15.15 ± 0.29a	21.93 ± 0.28a	14.32 ± 0.27a	99.79	69.54 ± 8.54a	26.45 ± 1.13i	55.43 ± 4.51
PT4	4.74 ± 0.05a	3.84 ± 0.08b	13.34 ± 0.20b	21.52 ± 0.37b	12.41 ± 0.21b	93.10	39.03 ± 1.26 cd	20.16 ± 1.30j	47.56 ± 1.00
PT5	4.63 ± 0.06b	3.41 ± 0.07c	11.75 ± 0.19c	21.28 ± 0.15b	12.33 ± 0.15b	88.12	28.84 ± 5.39efghi	28.81 ± 2.44hi	>60
PT6	3.33 ± 0.07ef	2.25 ± 0.09 h	7.45 ± 0.21 h	11.61 ± 0.35de	8.23 ± 0.22d	56.84	17.84 ± 1.19jkl	32.95 ± 0.51 fg	47.14 ± 1.24
PT7	3.30 ± 0.04f	1.67 ± 0.04 k	6.13 ± 0.14 k	10.18 ± 0.21 fg	6.53 ± 0.10 g	48.48	15.46 ± 3.52klm	22.87 ± 1.78j	>60
PT8	1.96 ± 0.04 m	0.75 ± 0.04o	2.82 ± 0.11o	6.55 ± 0.12j	2.19 ± 0.06 m	24.63	34.7 ± 2.81cde	49.29 ± 3.90bcd	>60
PT9	2.71 ± 0.06i	2.39 ± 0.17j	6.78 ± 0.14j	10.46 ± 0.12f	6.72 ± 0.22 g	50.85	32.88 ± 6.87edf	47.67 ± 4.52 cd	>60
PT10	3.42 ± 0.06e	2.18 ± 0.07ij	6.89 ± 0.09ij	12.48 ± 0.45c	7.97 ± 0.10de	56.54	22.34 ± 3.22hijk	30.10 ± 1.63gh	>60

In this study, there were large interspecies differences in TFC between PP and PT. For PP, TFC ranged from 0.83 to 1.83 mg RE/g dw, while those of PT were in the range of 0.75–4.14 mg RE/g dw. The mean TFC content of PT was higher than that of PP (*P* < 0.01) (Table 2). The Pearson correlation coefficient of TPC and TFC is 0.8, and the substances measured by them may mostly overlap. In previous studies, the TPC and PFC of pulp freeze-dried extracts of PP and PT showed significant differences. Compared with the results of these pulps, the TPC and TFC of PT kernels were also higher than that of PP. But the difference is smaller than between the flesh. The TPC and TFC of PP kernels were similar to that of their pulp, while the mean values of PT kernels and pulp were nearly ten times different(Z. Wang et al., 2023).

### 3.4 In vitro antioxidant activity

One of the negative effects of free radicals in the body is oxidative stress, which can contribute to aging and disease(Jomova et al., 2023). In this study, the antioxidant activity of two batches of cherry kernels was evaluated using DPPH and ABTS radical scavenging assays and FRAP. Trolox, a water-soluble vitamin E analog, was used as a standard. The results are presented in Table 2. Briefly, the DPPH radical scavenging capacity (*P* < 0.05), ABTS radical scavenging capacity (*P* < 0.01), and FRAP (*P* < 0.01) differed significantly among the species. PT kernels had the highest DPPH radical scavenging ability (ranging from 2.82 ± 0.11 to 15.15 ± 0.29 μmol TE/g dw), FRAP (ranging from 2.19 ± 0.06 to 14.32 ± 0.27 μmol TE/g dw) and ABTS radical scavenging ability (ranging from 6.55 ± 0.12 to 21.93 ± 0.28 μmol TE/g dw). On the other hand, PP kernels had the lowest DPPH radical scavenging ability (ranging from 4.29 ± 0.09 to 10.54 ± 0.25 μmol TE/g dw), FRAP (ranging from 3.89 ± 0.28 to 9.18 ± 0.09 μmol TE/g dw) and ABTS radical scavenging ability (ranging from 6.38 ± 0.38 to 12.58 ± 0.31 μmol TE/g dw).

The kernels of Prunus genus fruits are often discarded, but contain large amounts of multifunctional compounds such as flavonoids and anthocyanins and lipophilic compounds such as carotenoids, which have strong antioxidant capacity. The kernels of these two Prunus genus fruits are a good source of antioxidants due to their strong antioxidant properties, which may maybe rich in these compounds. However, the antioxidant activity of the kernels of these two Prunus genus fruits was lower than previously reported fruit pulp. Interestingly, albino species PT8 had the lowest antioxidant activity in both fruit and kernel(Z. Wang et al., 2023). Anthocyanins are one of the important compounds that determine the antioxidant capacity of plants. The lower antioxidant activity may be related to anthocyanin content.

Pearson correlation analysis showed that the correlation of the three antioxidant measurements was >0.9, indicating that the three methods of in vitro antioxidant analysis were similar(Fig. 8A). The correlation between TPC and TFC and the three antioxidant activity values was >0.8, indicating that phenolic compounds and flavonoids may be the main contributors to antioxidant capacity in vitro. However, due to the lack of sufficient specificity of the two content determination methods(Silva & Sirasa, 2018), compounds with high contributions to anti-oxidation need to be further identified.

ACI is a commonly used tool for integrating multiple indicators of antioxidant activity into a holistic approach(Lin et al., 2022). Since DPPH, FRAP, ABTS, TPC, and TFC are all commonly used methods to characterize in vitro antioxidant capacity, they were integrated into ACI (Table2).

### 3.5 Cell antioxidant activity(CAA) assay

CAA assays are more biologically relevant than chemical antioxidant assays and reflect the ability of chemicals to penetrate through cells against antioxidants. Nonpolar DCFH-DAcan penetrate the cell to acetylate into polar DCFH, and then AAPH enters the cell to oxidize DCFH into fluorescent DCF. antioxidants can reduce the fluorescence intensity. Therefore, the antioxidant capacity of the extract is inversely proportional to the fluorescence intensity of Caco-2 cells(Wolfe & Rui, 2007). This experiment only considers active compounds that act on the inside of cells, so they are immediately washed with PBS after the extraction solution interacts with the cells. According to CAA results (Table 2), both kernels extracts of PP and PT exhibited cellular antioxidant activity, inhibiting peroxyradical radical-induced oxidation, with CAA values of 8.82 ± 1.04 to 48.66 ± 1.17 μmol QE/100 g (PP) and 15.46 ± 3.52 to 69.54 ± 8.54 μmol QE/100 g (PT). The CAA average value of PT(32.232 ± 15.846 μmol QE/100 g) is greater than that of PP(23.315 ± 11.485 μmol QE/100 g)(*P* < 0.05).

The Pearson correlation coefficients of TPC and TFC with CAA were 0.60 and 0.55(*P* < 0.01), lower than the correlation with in vitro antioxidant activity (Fig. 8A). The possible reason is that all compounds can participate in the reaction in the solution system in vitro, and some substances do not enter the cell well. For example, in TPC, TFC, and three in vitro antioxidant assays, the lowest PT8 was found in the 10 batch PT samples, and ranked 4th in CAA assays. This may be because the anthocyanin compounds may not be able to enter the cell easily(Munagala et al., 2017). So the low content of PT8 anthocyanins will not result in lower CAA Other possible reasons are that different batches of samples have different types and contents of phenolic acids, flavonoids, and other compounds. According to the comparison of the antioxidant ability of different compounds to Caco-2cells measured before, different compounds have great differences. Ferulic acid, gallic acid and other compounds with strong antioxidant activity in vitro showed low or almost no cellular antioxidant activity(Wan et al., 2015b). Meanwhile, the Pearson's correlation coefficients of CAA and all three in vitro antioxidant activity test values were >0.5 (*P* < 0.05), which demonstrated that there was some difference between CAA and in vitro antioxidant activity assay values, but the same trend was observed.

### 3.6 Antiproliferative activity and cytotoxicity in PP and PT

Since high TPC, TFC and antioxidant activities were detected in both species of kernel extracts in previous experiments, the antiproliferative and cytotoxic activities of the cells of these extracts were further evaluated and the results are shown in Table 2. The lower median effective dose (IC_50_) value represented higher antiproliferative ability. Kernel extracts of both species exhibited potent antiproliferative activities in dose-dependent manners. IC_50_ values of PP and PT 32.69 ± 1.55 to 59.59 ± 1.70 and 20.16 ± 1.30 to 51.94 ± 1.71 mg/mL, respectively. The IC_50_ of PT was lower than that of PP(*P* < 0.01), so PT had a stronger antiproliferative ability.

The lowest CC_20_ was 40.79 ± 2.43 in the PP group and 47.14 ± 1.24 in the PT group. The CC_20_ of all the cherry kernels was >40 mg/mL. It was much lower than the concentration of 20 mg/mL used in the CAA test. At the same time, the toxicity of the extracts of all the samples had almost no effect on the CAA test due to the short duration of the action time of the CAA test. It is also noteworthy that the IC_50_values were higher than the CC_20_ values for all samples except PP5. This indicates that there was little cytotoxicity in the extracts at concentrations that produced strong antiproliferative activity, the anticancer activity of kernels was mainly attributed to antiproliferative effects rather than cytotoxicity. Pearson's correlation analysis showed that the antiproliferative activity had the highest negative correlation with TPC (−0.78), followed by the second highest negative correlation with TFC (−0.59) with CAA and in vitro antioxidant activity also showed significant negative correlation (*P* < 0.05) (Fig. 8A). This demonstrated that the antiproliferative capacity was mainly derived from phenolic compounds and proved that there was some synergistic effect between the different bioactivities.

### 3.7 Effect of kernel extracts on cycle arrest and apoptosis of CaCO-2 cells

**Fig. 6:**
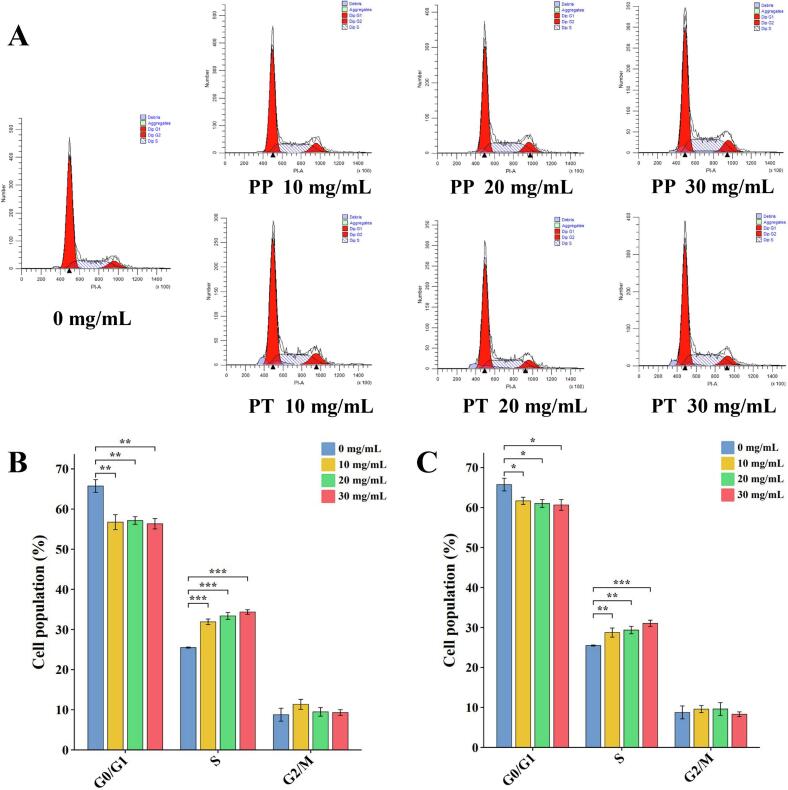
Cell-cycle analyses of Caco-2 cells exposed to extracts of cherry kernel at different concentrations for 72 h.(A) Representative histograms of DNA content in Caco-2cells treated for 72 h. Horizontal and vertical axes indicate the relative nuclear DNA content and number of cells, respectively.(B) The ratio of G0/G1, S and G2/M cell cycles after PP extract treatment(mean ± SD, *n* = 3).(C) The ratio of G0/G1, S and G2/M cell cycles after PT extract treatment(mean ± SD, n = 3).*, ** and *** represent significant correlations at the *P* ≤ 0.05, *P* ≤ 0.01, and *P* ≤ 0.001 levels, respectively. PP, *Prunus pseudocerasus*; PT, *Prunus tomentosa*.

According to the results of the CCK-8 experiment, the Caco-2 cells were treated with PP or PT extracts with concentrations of 10 mg/mL, 20 mg/mL, and 30 mg/mL, and then analyzed. As shown in Fig. 6A, compared with the blank group, it was found that the extracts of cherry kernel of both species at three concentrations of 10 mg/mL, 20 mg/m, and 30 mg/mL could effectively block the cycle of Caco-2cells in S phase, and the proportion of cell S phase increased with the increase of extract concentration. The proportion of the S phase in the three groups was significantly different from that in the blank group (*P* < 0.05) (Fig. 6B and C). These results indicated that both cherry kernel extracts could effectively interrupt the cell cycle progression of Caco-2cells at the S phase in a concentration-dependent manner.

**Fig. 7:**
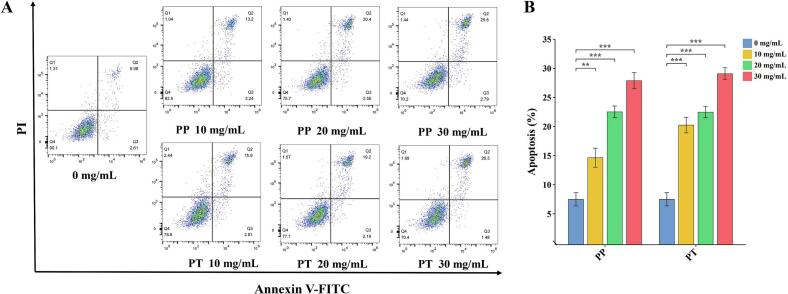
Cells apoptosisanalyses of Caco-2 cells exposed to extracts of cherry kernel at different concentrations for 72 h.(A) Caco-2 cell apoptosis. The percentage of apoptotic cells (both early and late apoptotic cells) was summarized according to flow cytometry spot maps after the cells were treated with samples.(B) The ratio apoptotic cells(mean ± SD, n = 3).*, ** and *** represent significant correlations at the *P* ≤ 0.05, *P* ≤ 0.01, and *P* ≤ 0.001 levels, respectively. PP, *Prunus pseudocerasus*; PT, *Prunus tomentosa*.

Caco-2cells were treated with extracts of cherry kernel at concentrations of 10 mg/mL, 20 mg/m, and 30 mg/mL, and apoptosis of Caco-2cells was obtained by flow cytometry. As shown in Fig. 7A and B, compared with the results of the blank group, it was found that the extract of cherry kernel of the two species at the concentration of 10 mg/mL, 20 mg/m and 30 mg/mL could effectively promote the apoptosis of Caco-2cells, and the apoptosis rate of the cells significantly increased with the increase of the concentration of the extract of cherry kernel of the two species.

### 3.8 Correlations of differential compounds with the biological activities

**Fig. 8:**
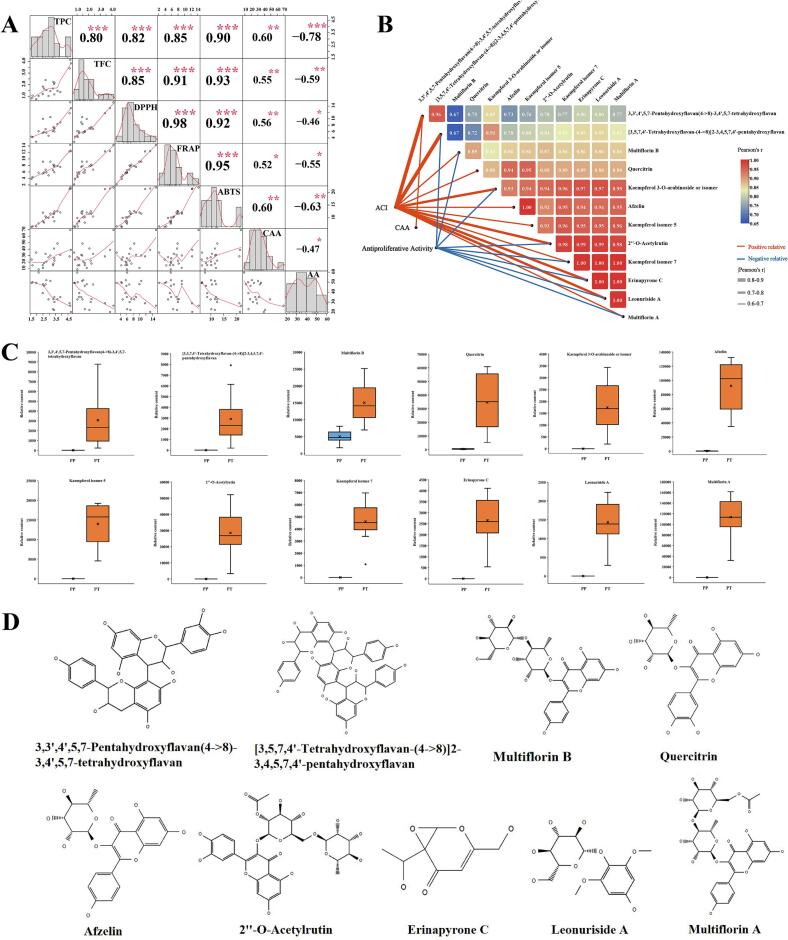
Correlation analysis between in vitro biological activity indicators (A).TPC, Total phenolic content; TFC, total flavonoid content; CAA, cellular antioxidant activity; AA, antiproliferative activity. *, ** and *** represent significant correlations at the *P* ≤ 0.05, *P* ≤ 0.01, and *P* ≤ 0.001 levels, respectively. Correlation map between differential compounds and ACI (TPC, TFC, DPPH, FRAP, ABTS), CAA, and antiproliferative activity (B). The thickness of the line represents the absolute value of the correlation coefficient, with orange representing a positive correlation and blue a negative correlation. Boxplot of Relative content of 12 key bioactive compounds(C).Possible structures of 9 compounds in 12 key bioactive compounds except for the unidentified isomers (D). (For interpretation of the references to color in this figure legend, the reader is referred to the web version of this article.)

Due to the composition of the chemicals, the content is the main determinant of the magnitude of the various biological activities. Consequently, Pearson's correlation tests was used to determine the relationship between biological activity and chemical composition. In order to screen for compounds with strong contributions to bioactivity, compounds with correlation |r| >0.6, *P* < 0.05 were selected. Fig. 8Bclearly showed that a total of 12 potential markers are positively associated with ACI, CAA, or antiproliferative activity, and all the correlations were statistically significant. Interestingly, all the screened compounds were positively correlated with ACI, and CAA or negatively correlated with IC_50_ values for antiproliferative activity. This indicated that all compounds with |r| ≥0.6, *P* < 0.05 are compounds with positive effects on biological activity. All of these compounds were positively correlated with ACI, 3,3′,4′,5,7-pentahydroxyflavan-(4- > 8)-3,4′,5,7-tetrahydroxyflavan was positively correlated with CAA, [3,5,7,4’-Tetrahydroxyflavan-(4- > 8)]2–3, 4,5,7,4′-pentahydroxyflavan, multiflorin B, kaempferol 3-O-arabinoside or isomer, 2”-O-Acetylrutin, kaempferol isomer 7, and erinapyrone C, leonuriside A, multiflorin A were negatively correlated with antiproliferative activity. The correlations among these 12 compounds were also all >0.6 (*P* < 0.05), showing content synergy. As Fig. 8C demonstrated the relative contents of the 12 compounds, it can be seen that allthese compounds were higher in PT than PP, and except for Multiflorin B, the other compounds were hardly contained in PP. In other words, those compounds that are specifically present in PT kernels are responsible for the generally higher biological activity of PT than PP.

These 12 potential markers included 8 Flavonoids, 1 Organooxygen compound,1 Pyrans, and 2 unidentified, the structures of 9 confirmed compounds were shown in Fig. 8D. Flavonoids are secondary metabolites that are distributed in almost all parts of all plants and have immunomodulatory, antimicrobial, anti-inflammatory, antiproliferative, and antioxidant functions due to their special structure (Shen et al., 2022). Eight of the 12 key bioactive compounds screened were flavonoids. 3,3′,4′,5,7-Pentahydroxyflavan(4- > 8)-3,4′,5,7-tetrahydroxyflavan and [3,5,7,4’-Tetrahydroxyflavan-(4- > 8)]] 2–3,4,5,7,4′-pentahydroxyflavan belong to the class of biflavonoids and polyflavonoids. Biflavonoids and polyflavonoids are flavonoids widely found in plants.Their structure is made up of two or more similar or dissimilar flavonoid units linked by C single C or C-O-C bonds, resulting in dimeric molecules or polymolecular, and there are numerous studies confirming the biological activity of these compounds(Menezes & Campos, 2021). Both compounds had the highest correlation with ACI, both exceeding 0.8, and the former was the only compound screened for positive correlation with CAA, while the latter was negatively correlated with antiproliferative activity. Multiflorin B, afzelin, quercitrin, and multiflorin A were identified from the kernel of PT in a previous study and have also been identified in the kernels of other Rosaceae, and been reported to have NO inhibition and antioxidant capacity, with quercitrin being reported to have the most potent DPPH radical scavenging activity(Kim et al., 2008). 2”-O -Acetylrutin and kaempferol 3-O-arabinoside or ismor are also flavonoids widely present in plants with various biological functions such as anticoagulant and anticancer(Sun et al., 2017; Yoshikawa et al., 2002). In addition, there are also two isomers of kaempferol with the same elemental composition as the standard Kaempferol, but with different retention times, which are also very likely to be flavonoids. The other two compounds are pyrans erinapyrone C and organooxygen compounds leonuriside A. The former is found in the genus Fungi with antiproliferative activity (Omolo et al., 2002)and the latter is found in Rhus parviflora fruits, Lonchocarpus bark with cytotoxic and antimicrobial activities as well as anti-inflammatory activity(Deskins et al., 2014; Shrestha et al., 2013). Of the 49 differential compounds, only 14 were flavonoids, while most of the key bioactive compounds screened were flavonoids, proving that the disparity in the content of certain flavonoids was the starring cause of the dominant antioxidant and antiproliferative activities.

Nevertheless, this study also discovered that kernels accumulate significant levels of cyanogenic glycosides, including amygdalin (D-mandelonitrile-β-D-gentiobioside) and (*S*)-2-Hydroxy-2-phenylacetonitrile *O*-b-D-allopyranoside(Supplementary data 2). Cyanogenic glycosides are mainly found in Rosaceae, but are also widely distributed in other plants, and consist mainly of α-hydroxynitriles derivatives(Kicel, 2020). Cyanogenic glycosides release hydrogen cyanide upon hydrolysis, which has been interpreted in previous studies as a defense mechanism against herbivores and pathogens(Tomishima et al., 2022). Moreover, cyanogenic glycosides have been suggested to be involved in seed germination(Gleadow & Møller, 2014). Cyanogenic glycosides can be toxic to humans, causing symptoms of cyanide poisoning like dizziness, diarrhea, nausea, and confusion if ingested excessively(Cressey & Reeve, 2019). Therefore, cyanogenic glycosides are considered to be anti-nutritionalcompounds.(Munekata et al., 2023). However, its positive bioactive effects include anti-inflammatory, antibacterial, antioxidant, and immunomodulatory effects(Barakat et al., 2022), as well as reported but unproven anticancer activity has also been widely reported (Spanoudaki et al., 2023).

## 4 Conclusion

This study is the first to comprehensively analyze the chemical composition and biological activities of kernels from two cherry species. A total of 193 compounds were identified and a total of 49 differential compounds screened. According to the biochemical indexes, both *Prunus tomentosa* and *Prunus pseudocerasus* had high biological activities, and the mean values of in vitro antioxidant activity, cellular antioxidant activity, and antiproliferative activity of *Prunus tomentosa* were higher than those of *Prunus pseudocerasus* (*P* < 0.05). Moreover, cytotoxicity analysis showed that kernel extracts were almost non-toxic at cellular antioxidant activity assay concentrations as well as at antiproliferative IC_50_ concentrationsand exhibited effective cell growth inhibition by inducing Caco-2 cells to undergo S phase arrest and apoptosis. Correlation analysis showed that 12 compounds had a positive effect on the biological activities of the kernel (|r| ≥0.6, *P* < 0.05), and 8 of these 12 compounds were flavonoids. Flavonoids are likely to be the main contributors to the observed biological activities of these kernels. In future studies, the in vitro biological activities of these flavonoids should be investigated by establishing animal models of kernels while paying attention to the possible toxicity effects of cyanogenic glycosides. It can be concluded that the kernels of both species can be used as a source of natural antioxidants and antiproliferative agents. Also, this study demonstrates a metabolomics strategy that can contribute to the rational utilization of food by-products.

## CRediT authorship contribution statement

**Ziwei Wang:** Writing – original draft. **Lin Li:** Methodology, Investigation. **Jiaqi Han:** Data curation. **Xinyu Bai:** Methodology. **Binbin Wei:** Validation, Supervision. **Ronghua Fan:** Writing – review & editing.

## Declaration of competing interest

The authors declare that they have no known competing financial interests or personal relationships that could have appeared to influence the work reported in this paper.

## Data Availability

Data will be made available on request.

## Appendix A Supplementary data

Supplementary material 1 Details of the identified compounds Supplementary material 1

Supplementary material 2 Common adduct ions for queretin(A), identification of queretin(B). Supplementary material 2

Supplementary material 3 Compound 3.90_609.1462m/z was identified as Rutin by rutin standard and 4.42_609.1470m/z was identified as Rutin isomer. Supplementary material 3
