# Supplementary material for: Combined metabolomics and bioactivity assays kernelby-productsof two native Chinese cherry species: The sources of bioactive nutraceutical compounds
Source: Food Chem X. 2024 Jul 5;23:101625. doi: 10.1016/j.fochx.2024.101625 (PMC11296007; doi:10.1016/j.fochx.2024.101625)
Supplement: Supplementary file 1 — Supplementary material 1 Details of the identified compounds [file mmc1.docx]

**Table 1.** Details of the identified compounds.

| **Compound ID** | **HMDB.ID** | **Name** | **Formula** | **Mass Error (ppm)** | **PP** | **PT** | **Super.class** | **Class** | **Differential compounds** |
| --- | --- | --- | --- | --- | --- | --- | --- | --- | --- |
| 0.50_146.1059n | HMDB0000182 | L-Lysine | C6H14N2O2 | 2.49 | + | + | Organic acids and derivatives | Carboxylic acids and derivatives |  |
| 0.50_173.1036m/z | HMDB0000517 | L-Arginine | C6H14N4O2 | -4.63 | + | + | Organic acids and derivatives | Carboxylic acids and derivatives | + |
| 0.55_181.0707m/z | HMDB0000765 or HMDB0000247 | Hexitol or Sorbitol | C6H14O6 | -5.92 | + | + | Organic oxygen compounds | Organooxygen compounds | + |
| 0.55_132.0524n | HMDB0000168 | Asparagine | C4H8N2O3 | -8.45 | + | + | Organic acids and derivatives | Carboxylic acids and derivatives |  |
| 0.55_146.0681n | HMDB0000641 | L-Glutamine | C5H10N2O3 | -7.28 | + | + | Organic acids and derivatives | Carboxylic acids and derivatives |  |
| 0.55_130.0495m/z | HMDB0000267 | L-Pyroglutamic acid | C5H7NO3 | -2.92 | + | + | Organic acids and derivatives | Carboxylic acids and derivatives | + |
| 0.55_132.0294m/z | HMDB0000191 | L-Aspartic acid | C4H7NO4 | -6.53 | + | + | Organic acids and derivatives | Carboxylic acids and derivatives |  |
| 0.55_147.0522n | HMDB0000148 | L-Glutamic acid | C5H9NO4 | -6.55 | + | + | Organic acids and derivatives | Carboxylic acids and derivatives | + |
| 0.55_342.1159n | HMDB0000258 | Sucrose | C12H22O11 | -0.79 | + | + | Organic oxygen compounds | Organooxygen compounds |  |
| 0.56_119.0580n | HMDB0000167 | L-Threonine | C4H9NO3 | -1.74 | + |  | Organic acids and derivatives | Carboxylic acids and derivatives |  |
| 0.57_116.0700m/z | HMDB0000162 | L-Proline | C5H9NO2 | -5.50 | + | + | Organic acids and derivatives | Carboxylic acids and derivatives |  |
| 0.57_196.0577n | HMDB0000625 | Gluconic acid | C6H12O7 | -3.05 | + | + | Organic oxygen compounds | Organooxygen compounds |  |
| 0.58_236.0767m/z | HMDB0037848 | N-(1-Deoxy-1-fructosyl)glycine | C8H15NO7 | -3.58 | + | + | Organic oxygen compounds | Organooxygen compounds |  |
| 0.60_179.0551m/z | HMDB0000122 | beta-D-Glucose | C6H12O6 | -5.45 | + | + | Organic oxygen compounds | Organooxygen compounds |  |
| 0.60_239.0764m/z | HMDB0029954 | D-glycero-L-galacto-Octulose | C8H16O8 | -3.45 | + | + | Organic oxygen compounds | Organooxygen compounds |  |
| 0.61_169.0608n | HMDB0304046 | 2,6-diamino-4-hydroxy-5-formamidopyrimidine | C5H7N5O2 | 4.68 | + | + | Organic nitrogen compounds | Organonitrogen compounds | + |
| 0.61_175.0240m/z | HMDB0000044 | L-Ascorbic acid | C6H8O6 | -4.69 | + | + | Organoheterocyclic compounds | Dihydrofurans |  |
| 0.62_150.0595m/z | HMDB0000696 | l-Methionine | C5H11NO2S | 8.15 | + |  | Organic acids and derivatives | Carboxylic acids and derivatives |  |
| 0.62_115.0027m/z | HMDB0000134 | Fumaric acid | C4H4O4 | -8.61 | + | + | Organic acids and derivatives | Carboxylic acids and derivatives |  |
| 0.80_308.0916m/z | HMDB0000125 | Glutathione | C10H17N3O6S | 1.70 | + | + | Organic acids and derivatives | Carboxylic acids and derivatives |  |
| 0.80_145.0133m/z | HMDB0000208 | 2-Oxoglutaric acid | C5H6O5 | -6.66 | + |  | Organic acids and derivatives | Keto acids and derivatives |  |
| 0.81_301.1035n | HMDB0304096 | 3''-deamino-3''-oxonicotianamine | C12H17N2O7- | -0.30 | + | + | Organic acids and derivatives | Carboxylic acids and derivatives |  |
| 0.81_111.0079m/z | HMDB0000617 | 2-Furancarboxylic acid | C5H4O3 | -8.09 | + | + | Organoheterocyclic compounds | Furans | + |
| 0.81_192.0266n | HMDB0000094 | Citric acid | C6H8O7 | -2.26 | + | + | Organic acids and derivatives | Carboxylic acids and derivatives | + |
| 0.85_132.1018m/z | HMDB0000172 | L-Isoleucine | C6H13NO2 | -0.43 | + | + | Organic acids and derivatives | Carboxylic acids and derivatives |  |
| 1.09_327.1331n | HMDB0037846 | N-(1-Deoxy-1-fructosyl)phenylalanine | C15H21NO7 | 4.11 | + |  | Organic acids and derivatives | Carboxylic acids and derivatives |  |
| 1.18_495.1496m/z | HMDB0039357 | Dihydroprudomenin | C23H26O12 | -0.16 | + | + | Phenylpropanoids and polyketides | Flavonoids |  |
| 1.21_164.0709m/z | HMDB0000159 | L-Phenylalanine | C9H11NO2 | -4.97 | + | + | Organic acids and derivatives | Carboxylic acids and derivatives | + |
| 1.22_118.0649m/z | HMDB0034171 | Benzeneacetonitrile | C8H7N | -1.77 | + | + | Benzenoids | Benzene and substituted derivatives |  |
| 1.22_148.0525n | HMDB0000567 | Cinnamic acid | C9H8O2 | 0.29 | + | + | Phenylpropanoids and polyketides | Cinnamic acids and derivatives | + |
| 1.27_218.1029m/z | HMDB0000210 | Pantothenic acid | C9H17NO5 | -2.35 | + | + | Organic oxygen compounds | Organooxygen compounds |  |
| 1.29_358.1145m/z | HMDB0029452 | L-DOPA 3'-glucoside | C15H21NO9 | 0.44 | + | + | Organic acids and derivatives | Carboxylic acids and derivatives |  |
| 1.64_649.1985m/z | HMDB0034682 | cis-Ferulic acid [arabinosyl-(1->3)-[glucosyl-(1->6)]-glucosyl] ester | C27H38O18 | -0.09 | + |  | Organic oxygen compounds | Organooxygen compounds |  |
| 1.82_298.0981m/z | HMDB0001173 | 5'-Methylthioadenosine | C11H15N5O3S | 4.18 | + |  | Nucleosides, nucleotides, and analogues | 5'-deoxyribonucleosides |  |
| 1.83_594.2163n | HMDB0034955 | Zizybeoside II | C25H38O16 | 0.52 | + | + | Organic oxygen compounds | Organooxygen compounds | + |
| 1.85_353.0874m/z | HMDB0003164 | Chlorogenic acid isomer1 | C16H18O9 | -1.06 | + |  | Organic oxygen compounds | Organooxygen compounds |  |
| 1.96_313.0927m/z | HMDB0038333 or HMDB0038335 | 2-Hydroxyphenylacetic acid O-b-D-glucoside or (S)-Mandelic acid O-beta-D-Glucopyranoside | C14H18O8 | -0.58 | + | + | Organic oxygen compounds | Organooxygen compounds | + |
| 1.97_314.1006n | HMDB0034060 | 6-O-Acetylarbutin | C14H18O8 | 1.37 | + | + | Organic oxygen compounds | Organooxygen compounds | + |
| 2.00_789.2473m/z | HMDB0304525 | UDP-3-O-(3-hydroxymyristoyl)-alpha-D-glucosamine | C29H50N3O18P2- | -2.39 | + |  | Nucleosides, nucleotides, and analogues | Pyrimidine nucleotides |  |
| 2.24_432.1638n | HMDB0041515 | Benzyl gentiobioside | C19H28O11 | 1.37 | + | + | Organic oxygen compounds | Organooxygen compounds | + |
| 2.25_432.1623n | HMDB0034954 | Zizybeoside I | C19H28O11 | -2.10 | + | + | Organic oxygen compounds | Organooxygen compounds | + |
| 2.28_445.1350m/z | HMDB0035007 | Lucuminic acid | C19H26O12 | -0.41 | + |  | Organic oxygen compounds | Organooxygen compounds | + |
| 2.32_293.1237m/z | HMDB0031693 | Ethyl (S)-3-hydroxybutyrate glucoside | C12H22O8 | -1.74 | + | + | Lipids and lipid-like molecules | Fatty Acyls |  |
| 2.36_405.0799m/z | HMDB0003572 | Rosmarinic acid | C18H16O8 | -7.85 |  | + | Phenylpropanoids and polyketides | Cinnamic acids and derivatives |  |
| 2.45_353.0873m/z | HMDB0003164 | Chlorogenic acid | C16H18O9 | -1.35 | + |  | Unidentified | Unidentified |  |
| 2.46_191.0554m/z | HMDB0003072 | Quinic acid | C7H12O6 | -3.92 |  |  | Organic oxygen compounds | Organooxygen compounds |  |
| 2.60_457.1592n | HMDB0035030 | Amygdalin | C20H27NO11 | 1.74 | + | + | Organic oxygen compounds | Organooxygen compounds |  |
| 2.61_162.0530n | HMDB0041561 | D-1,5-Anhydrofructose | C6H10O5 | 1.06 | + | + | Organoheterocyclic compounds | Oxanes |  |
| 2.68_151.0392m/z | MDB0000440 | 3-Hydroxyphenylacetic acid | C8H8O3 | -5.50 | + | + | Benzenoids | Phenols |  |
| 2.77_577.1356m/z | HMDB0029754 or HMDB0033974 or HMDB0033973 or HMDB0013690 or 130556^a^ | Procyanidin B1 or Procyanidin B3 or Procyanidin B2 or Procyanidin B4 or Procyanidin B | C30H26O12 | 0.79 | + | + | Phenylpropanoids and polyketides | Flavonoids |  |
| 2.80_490.1686n | HMDB0032743 | Phloroacetophenone 6'-[xylosyl-(1->6)-glucoside] | C21H30O13 | -0.10 | + | + | Organic oxygen compounds | Organooxygen compounds |  |
| 2.80_402.1528n | HMDB0041514 or HMDB0303056 | Benzyl O-[arabinofuranosyl-(1->6)-glucoside] or Benzyl 6-O-beta-D-apiofuranosyl-beta-D-glucoside | C18H26O10 | 0.44 | + | + | Organic oxygen compounds | Organooxygen compounds | + |
| 2.85_237.0633n | HMDB0034251 | N-Benzoylaspartic acid | C11H11NO5 | -1.74 | + |  | Organic acids and derivatives | Carboxylic acids and derivatives |  |
| 2.85_284.0892n | HMDB0034618 | 2-O-Benzoyl-D-glucose | C13H16O7 | -1.43 | + |  | Organic oxygen compounds | Organooxygen compounds |  |
| 2.86_307.0806m/z | HMDB0038361 | Epigallocatechin | C15H14O7 | -2.15 | + |  | Phenylpropanoids and polyketides | Flavonoids |  |
| 2.90_446.1772n | HMDB0039253 | Crosatoside B | C20H30O11 | -3.70 | + | + | Organic oxygen compounds | Organooxygen compounds | + |
| 2.92_994.2550n | HMDB0041267 | Quercetin 3-(6''-sinapoylsophorotrioside) | C44H50O26 | -4.05 | + |  | Phenylpropanoids and polyketides | Flavonoids | + |
| 2.96_284.0891n | HMDB0038492 or HMDB0303039 or HMDB0303674 | D-Vacciniin or 6-O-Benzoyl-alpha-D-glucose or Vacciniin | C13H16O7 | -1.91 | + | + | Benzenoids | Benzene and substituted derivatives |  |
| 3.01_587.2457m/z | HMDB0038221 | Pentigetide | C22H36N8O11 | 4.40 | + | + | Phenylpropanoids and polyketides | Isoflavonoids | + |
| 3.07_290.0789n | HMDB0033783 | Marshrin | C15H14O6 | -0.63 | + | + | Phenylpropanoids and polyketides | Coumarins and derivatives |  |
| 3.07_771.1987m/z | 10190763^a^ | Quercetin 3-O-rutinoside-7-O-glucoside | C33H40O21 | -0.27 | + |  | Phenylpropanoids and polyketides | Flavonoids |  |
| 3.08_291.0875m/z | HMDB0001871 or HMDB0002780 | Epicatechin or Catechin | C15H14O6 | 4.13 | + | + | Phenylpropanoids and polyketides | Flavonoids |  |
| 3.10_327.1082m/z | HMDB0038334 | Dihydromelilotoside | C15H20O8 | -0.94 | + |  | Organic oxygen compounds | Organooxygen compounds | + |
| 3.12_458.1426n | HMDB0041336 | 7-Hydroxy-4-methylphthalide O-[arabinosyl-(1->6)-glucoside] | C20H26O12 | 0.28 | + |  | Organic oxygen compounds | Organooxygen compounds |  |
| 3.12_337.0928m/z | HMDB0301709 or HMDB0029681 or HMDB0301710 | 5-p-Coumaroylquinic acid or 3-p-Coumaroylquinic acid or 4-p-Coumaroylquinic acid | C16H18O8 | -0.18 |  | + | Organic oxygen compounds | Organooxygen compounds |  |
| 3.16_427.1477n | HMDB0029900 | Lucuminoside | C19H25NO10 | -0.43 | + | + | Organic oxygen compounds | Organooxygen compounds |  |
| 3.23_866.2054n | HMDB0038370 | Procyanidin C1 | C45H38O18 | -0.46 | + | + | Phenylpropanoids and polyketides | Flavonoids |  |
| 3.27_131.0705m/z | HMDB0000746 | (+)-2-Hydroxyisocaproic acid | C6H12O3 | -6.82 | + |  | Lipids and lipid-like molecules | Fatty Acyls |  |
| 3.29_295.1058n | HMDB0039961 | (S)-2-Hydroxy-2-phenylacetonitrile O-b-D-allopyranoside | C14H17NO6 | 0.57 | + | + | Organic oxygen compounds | Organooxygen compounds | + |
| 3.29_610.1916m/z | HMDB0303649 | Peonidin 3-rutinoside | C28H33O15+ | 3.87 | + | + | Phenylpropanoids and polyketides | Flavonoids | + |
| 3.31_625.1468m/z | 7215-44-3^b^ | Quercetin 3-sophoroside | C27H30O17 | 9.24 | + | + | Phenylpropanoids and polyketides | Flavonoids |  |
| 3.35_561.1403m/z | HMDB0037662 | 3,3',4',5,7-Pentahydroxyflavan(4->8)-3,4',5,7-tetrahydroxyflavan | C30H26O11 | 0.07 |  | + | Phenylpropanoids and polyketides | Flavonoids | + |
| 3.42_756.2112n | HMDB0302425 | Kaempferol 3-O-rhamnodiglucoside | C33H40O20 | -0.16 | + |  | Phenylpropanoids and polyketides | Flavonoids | + |
| 3.48_461.1660m/z | HMDB0039233 | Verbasoside | C20H30O12 | -0.87 | + | + | Organic oxygen compounds | Organooxygen compounds |  |
| 3.55_441.1632n | HMDB0032803 | Mandelonitrile rutinoside | C20H27NO10 | -0.68 | + | + | Organic oxygen compounds | Organooxygen compounds |  |
| 3.61_475.1819m/z | HMDB0035635 | Kanokoside A | C21H32O12 | -0.44 | + |  | Organic acids and derivatives | Carboxylic acids and derivatives |  |
| 3.67_290.0899n | HMDB0038666 | L-N-(1H-Indol-3-ylacetyl)aspartic acid | C14H14N2O5 | -1.36 | + |  | Organic acids and derivatives | Carboxylic acids and derivatives | + |
| 3.69_209.0448m/z | HMDB0001713 or HMDB0030677 | m-Coumaric acid or p-Coumaric acid | C9H8O3 | -4.31 | + | + | Phenylpropanoids and polyketides | Cinnamic acids and derivatives |  |
| 3.71_385.1137m/z | 5280406^a^ | Sinapoylglucose | C17H22O10 | -0.72 | + |  | Phenylpropanoids and polyketides | Cinnamic acids and derivatives |  |
| 3.79_213.0750m/z | HMDB0033835 | Propyl gallate | C10H12O5 | -3.48 | + |  | Benzenoids | Benzene and substituted derivatives |  |
| 3.86_481.1333m/z | HMDB0036634 | Phloridzin | C21H24O10 | -4.24 |  |  | Phenylpropanoids and polyketides | Flavonoids |  |
| 3.86_312.1203n | HMDB0031724 | Moringyne | C15H20O7 | -1.83 | + |  | Organic oxygen compounds | Organooxygen compounds |  |
| 3.86_712.2215n | HMDB0041149 | Glucoliquiritin apioside | C32H40O18 | 0.01 | + |  | Phenylpropanoids and polyketides | Flavonoids |  |
| 3.89_597.1799m/z | HMDB0302619 | Leucopelargonidin 3-O-alpha-L-rhamno-beta-D-glucopyranoside | C27H34O15 | -4.39 | + |  | Phenylpropanoids and polyketides | Flavonoids |  |
| 3.89_207.1029m/z | HMDB0034122 | Aceteugenol | C12H14O3 | 6.52 | + |  | Benzenoids | Phenol esters |  |
| 3.90_609.1462m/z | HMDB0003249 | Rutin | C27H30O16 | 0.22 | + | + | Phenylpropanoids and polyketides | Flavonoids |  |
| 4.01_833.2081m/z | HMDB0039144 | [3,5,7,4'-Tetrahydroxyflavan-(4->8)]2-3,4,5,7,4'-pentahydroxyflavan | C45H38O16 | -0.68 |  | + | Phenylpropanoids and polyketides | Flavonoids | + |
| 4.03_624.1681m/z | 47851-83-2^b^ | Peonidin 3,5-di-O-glucoside | C28H33O16+ | -2.34 | + | + | Phenylpropanoids and polyketides | Flavonoids |  |
| 4.04_174.0550m/z | HMDB0000197 | Indole-3-acetic acid | C10H9NO2 | -6.25 |  |  | Organoheterocyclic compounds | Indoles and derivatives |  |
| 4.12_463.0881m/z | HMDB0030775 | Hyperoside isomer 1 | C21H20O12 | -0.21 | + | + | Unidentified | Unidentified |  |
| 4.15_413.1455m/z | HMDB0032600 | Ptelatoside A | C19H26O10 | 0.51 | + |  | Organic oxygen compounds | Organooxygen compounds | + |
| 4.15_227.0545m/z | HMDB0012199 | Chorismate | C10H10O6 | -2.37 | + | + | Organic acids and derivatives | Carboxylic acids and derivatives |  |
| 4.15_306.0963n | HMDB0029891 | Starch acetate | C12H18O9 | 4.13 | + |  | Organic acids and derivatives | Carboxylic acids and derivatives |  |
| 4.27_863.1832m/z | HMDB0037673 | Cinnamtannin D1 | C45H36O18 | 0.31 | + |  | Phenylpropanoids and polyketides | Flavonoids |  |
| 4.32_433.0773m/z | HMDB0030860 | Avicularin | C20H18O11 | -0.88 |  | + | Phenylpropanoids and polyketides | Flavonoids |  |
| 4.33_594.1590n | HMDB0303597 | Multiflorin B | C27H30O15 | 0.91 | + | + | Phenylpropanoids and polyketides | Flavonoids | + |
| 4.33_287.0568m/z | HMDB0005801 | Kaempferol isomer 1 | C15H10O6 | 6.40 | + | + | Unidentified | Unidentified |  |
| 4.42_609.1470m/z | HMDB0003249 | Rutin isomer1 | C27H30O16 | 1.45 |  | + | Unidentified | Unidentified | + |
| 4.42_302.0435n | HMDB0005794 | Quercetin isomer 1 | C15H10O7 | 2.87 |  | + | Unidentified | Unidentified | + |
| 4.48_755.2044m/z | 21676298^a^ | Kaempferol 3-O-rutinoside 7-O-glucoside | C33H40O20 | 0.48 |  | + | Phenylpropanoids and polyketides | Flavonoids |  |
| 4.48_434.1204n | 529-55-5^b^ | Prunin | C21H22O10 | -1.99 | + | + | Phenylpropanoids and polyketides | Flavonoids |  |
| 4.52_522.2100n | 220745-70-0^b^ | Glochidiobioside | C26H34O11 | -0.25 | + | + | Phenylpropanoids and polyketides | 2-arylbenzofuran flavonoids |  |
| 4.53_433.0784m/z | HMDB0037927 | Quercetin 7-xyloside | C20H18O11 | 1.78 |  | + | Phenylpropanoids and polyketides | Flavonoids |  |
| 4.57_287.0566m/z | HMDB0005801 | Kaempferol isomer 2 | C15H10O6 | 5.42 |  | + | Unidentified | Unidentified |  |
| 4.58_461.0724m/z | HMDB0240541 | Luteolin 7-O-glucuronide | C21H18O12 | -0.34 |  |  | Phenylpropanoids and polyketides | Flavonoids |  |
| 4.63_448.1010n | HMDB0033751 | Quercitrin | C21H20O11 | 0.92 | + | + | Phenylpropanoids and polyketides | Flavonoids | + |
| 4.73_463.0881m/z | HMDB0030775 | Hyperoside isomer 2 | C21H20O12 | -0.20 | + |  | Unidentified | Unidentified |  |
| 4.78_427.1605m/z | HMDB0034447 | Ptelatoside B | C20H28O10 | -1.09 | + |  | Organic oxygen compounds | Organooxygen compounds |  |
| 4.86_449.1083m/z | HMDB0029544 | Eriodictyol 7-O-glucoside | C21H22O11 | -1.45 |  | + | Benzenoids | Benzene and substituted derivatives |  |
| 4.88_613.1777m/z | 1607025-56-8^b^ | Tomenside C | C27H34O16 | 0.49 | + |  | Phenylpropanoids and polyketides | Cinnamic acids and derivatives | + |
| 4.89_309.0979m/z | HMDB0040692 | 4R,5R,6S-Trihydroxy-2-hydroxymethyl-2-cyclohexen-1-one 6-(2-hydroxy-6-methylbenzoate) | C15H16O7 | 3.39 | + |  | Benzenoids | Benzene and substituted derivatives |  |
| 4.89_287.0553m/z | HMDB0005801 | Kaempferol isomer 3 | C15H10O6 | 0.83 |  | + | Unidentified | Unidentified | + |
| 4.89_594.1577n | HMDB0302426 | Kaempferol 3-O-rutinoside | C27H30O15 | -1.36 |  | + | Phenylpropanoids and polyketides | Flavonoids | + |
| 4.90_303.0519m/z | HMDB0005794 | Quercetin isomer 2 | C15H10O7 | 6.48 |  | + | Unidentified | Unidentified |  |
| 4.92_533.0931m/z | HMDB0301681 | Luteolin 7-malonylglucoside | C24H22O14 | -1.10 |  |  | Phenylpropanoids and polyketides | Flavonoids |  |
| 4.99_579.1717m/z | HMDB0002927 | Naringin | C27H32O14 | -0.33 | + |  | Phenylpropanoids and polyketides | Flavonoids |  |
| 5.02_613.1780m/z | 1446205-86-2^b^ | Mumeose C | C27H34O16 | 0.98 | + |  | Phenylpropanoids and polyketides | Cinnamic acids and derivatives |  |
| 5.05_417.0823m/z | HMDB0029261 or HMDB0029502 or HMDB0301957 or HMDB0302329 or HMDB0303587 | Kaempferol 3-O-arabinoside or Kaempferol 3-alpha-L-arabinofuranoside or Kaempferol 3-alpha-D-arabinopyranoside or Kaempferol 3-O-beta-D-xylofuranoside or Juglanin | C20H18O10 | -0.93 |  | + | Phenylpropanoids and polyketides | Flavonoids | + |
| 5.05_287.0569m/z | HMDB0005801 | Kaempferol isomer 4 | C15H10O6 | 6.57 |  | + | Unidentified | Unidentified |  |
| 5.08_643.1879m/z | 1609630-41-2^b^ | Tomenside D | C28H36O17 | -0.09 | + | + | Organic acids and derivatives | Carboxylic acids and derivatives |  |
| 5.15_505.0985m/z | HMDB0029271 or HMDB0037366 or HMDB0038465 or HMDB0039923 | Quercetin 3-O-(6''-acetyl-glucoside) or Quercetin 3-(6''-acetylglucoside) or Glyphoside or 4''-O-Acetylmyricitrin | C23H22O13 | -0.43 | + | + | Phenylpropanoids and polyketides | Flavonoids |  |
| 5.17_227.0341m/z | HMDB0013695 | Urolithin A | C13H8O4 | -3.68 |  | + | Phenylpropanoids and polyketides | Coumarins and derivatives |  |
| 5.17_432.1059n | 5316673^a^ | Afzelin | C21H20O10 | 0.67 | + | + | Phenylpropanoids and polyketides | Flavonoids | + |
| 5.17_287.0551m/z | HMDB0005801 | Kaempferol isomer 5 | C15H10O6 | 0.20 |  | + | Unidentified | Unidentified | + |
| 5.18_709.2350m/z | HMDB0303777 | 2'-(4-Hydroxyphenylacetyl)-6'-(4-hydroxy-3-methylbutanoyl)-phloroacetophenone 4'-[rhamnosyl-(1->2)-glucoside] | C33H42O17 | 0.05 |  |  | Lipids and lipid-like molecules | Prenol lipids |  |
| 5.20_610.1542n | HMDB0003249 | Rutin isomer 2 | C27H30O16 | 1.41 |  | + | Unidentified | Unidentified |  |
| 5.20_301.0343m/z | HMDB0005794 | Quercetin isomer 3 | C15H10O7 | -3.46 |  | + | Unidentified | Unidentified |  |
| 5.20_652.1628n | HMDB0039929 | 2''-O-Acetylrutin | C29H32O17 | -1.76 |  | + | Phenylpropanoids and polyketides | Flavonoids | + |
| 5.40_681.1669m/z | HMDB0041232 | 8-Hydroxyhesperetin 7-[6-acetylglucosyl-(1->2)-glucoside] | C30H34O18 | -0.51 |  | + | Lipids and lipid-like molecules | Prenol lipids |  |
| 5.40_285.0396m/z | HMDB0005801 | Kaempferol isomer 6 | C15H10O6 | -3.15 |  | + | Unidentified | Unidentified |  |
| 5.40_288.0607m/z | HMDB0002708 | Cyanidin | C15H11O6+ | -7.38 |  | + | Phenylpropanoids and polyketides | Flavonoids |  |
| 5.56_577.1563m/z | HMDB0037349 or HMDB0038848 | Apigenin 7-O-rutinoside or Apigenin 7-O-neohesperidoside | C27H30O14 | 0.08 |  | + | Phenylpropanoids and polyketides | Flavonoids |  |
| 5.59_489.1035m/z | HMDB0301688 | Quercetin 3-O-acetyl-rhamnoside | C23H22O12 | -0.63 | + | + | Phenylpropanoids and polyketides | Flavonoids |  |
| 5.64_229.0497m/z | HMDB0039141 | Wyeronic acid | C13H10O4 | -3.95 |  | + | Lipids and lipid-like molecules | Fatty Acyls |  |
| 5.64_285.0399m/z | HMDB0005801 | Kaempferol isomer 7 | C15H10O6 | -2.01 |  | + | Unidentified | Unidentified | + |
| 5.64_186.0529n | HMDB0041026 | Erinapyrone C | C8H10O5 | 0.60 |  | + | Organoheterocyclic compounds | Pyrans | + |
| 5.64_332.1119n | HMDB0031721 | Leonuriside A | C14H20O9 | 3.45 |  | + | Organic oxygen compounds | Organooxygen compounds | + |
| 5.64_329.0712m/z | HMDB0033622 | Cabbage identification factor 2 | C15H12N4O3S | 2.87 | + | + | Lipids and lipid-like molecules | Prenol lipids |  |
| 5.64_636.1675n | HMDB0303598 | Multiflorin A | C29H32O16 | -2.36 |  | + | Phenylpropanoids and polyketides | Flavonoids | + |
| 5.64_974.2348m/z | HMDB0301905 | Malvidin 3-(6''-p-coumarylglucoside) 5-dimalonylglucoside | C44H45O25+ | 2.59 |  | + | Phenylpropanoids and polyketides | Flavonoids |  |
| 5.70_595.1672m/z | HMDB0255521 or HMDB0302534 | Neoeriocitrin or Isoeriocitrin | C27H32O15 | 0.60 | + |  | Phenylpropanoids and polyketides | Flavonoids | + |
| 5.74_536.1898n | HMDB0240735 | Isolariciresinol glucuronide | C26H32O12 | 0.73 | + | + | Lignans, neolignans and related compounds | Lignan glycosides | + |
| 5.82_417.1192m/z | HMDB0037490 | Neoliquiritin | C21H22O9 | 0.25 | + | + | Phenylpropanoids and polyketides | Flavonoids | + |
| 5.82_418.1262n | HMDB0029520 | Liquiritin | C21H22O9 | -0.37 | + |  | Phenylpropanoids and polyketides | Flavonoids |  |
| 5.83_248.0696n | HMDB0029971 | Coriandrone E | C13H12O5 | 4.68 | + |  | Organoheterocyclic compounds | Benzopyrans |  |
| 5.83_267.0871m/z | HMDB0251297 | Dihydrocitrinone | C13H14O6 | 3.12 | + |  | Benzenoids | Benzene and substituted derivatives |  |
| 5.83_532.1584n | HMDB0041209 | Sesaminol glucoside | C26H28O12 | 0.66 |  |  | Lignans, neolignans and related compounds | Lignan glycosides |  |
| 5.89_459.0931m/z | HMDB0041741 or HMDB0246212 or HMDB0259901 | Glycitein 7-O-glucuronide or Oroxylin A glucoronide or Wogonoside | C22H20O11 | -0.46 |  | + | Unidentified | Unidentified |  |
| 5.94_457.1138m/z | HMDB0030689 | 6''-O-Acetyldaidzin | C23H22O10 | -0.39 | + |  | Phenylpropanoids and polyketides | Isoflavonoids |  |
| 5.96_473.1087m/z | HMDB0039746 or HMDB0039747 or HMDB0039748 | 4''-O-Acetylafzelin or 3''-O-Acetylafzelin or Kaempferol 3-(2''-acetylrhamnoside) | C23H22O11 | -0.42 |  | + | Unidentified | Unidentified |  |
| 6.41_328.2249n | HMDB0035919 | Corchorifatty acid F | C18H32O5 | -0.27 | + | + | Lipids and lipid-like molecules | Fatty Acyls | + |
| 6.47_431.1346m/z | 115799-14-9^b^ | (2S)-5-(beta-D-Glucopyranosyloxy)-2,3-dihydro-7-methoxy-2-phenyl-4H-1-benzopyran-4-one | C22H24O9 | -0.29 |  |  | Phenylpropanoids and polyketides | Flavonoids |  |
| 6.49_271.0604m/z | 548-82-3^b^ | Pinobanksin | C15H12O5 | -3.05 | + |  | Phenylpropanoids and polyketides | Flavonoids |  |
| 6.53_269.0444m/z | HMDB0002124 | Apigenin | C15H10O5 | -4.10 | + |  | Phenylpropanoids and polyketides | Flavonoids |  |
| 6.63_393.1188m/z | HMDB0038066 | Garcimangosone D | C19H20O9 | 1.97 | + |  | Organic oxygen compounds | Organooxygen compounds |  |
| 6.65_445.1147m/z | HMDB0002219 | Glycitin | C22H22O10 | 1.60 | + | + | Phenylpropanoids and polyketides | Isoflavonoids |  |
| 6.75_357.0607m/z | HMDB0029272 | 5,4'-Dihydroxy-3,3'-dimethoxy-6:7-methylenedioxyflavone | C18H14O8 | -2.44 | + | + | Phenylpropanoids and polyketides | Flavonoids |  |
| 6.78_401.0874m/z | HMDB0303810 | Apigenin 7-arabinoside | C20H18O9 | -1.06 | + | + | Phenylpropanoids and polyketides | Flavonoids |  |
| 6.79_399.1315n | HMDB0040552 | Adlumidiceine | C21H21NO7 | -0.82 | + | + | Phenylpropanoids and polyketides | Stilbenes |  |
| 6.82_205.0862m/z | HMDB0302558 | Valencic acid | C12H14O3 | -3.82 | + |  | Benzenoids | Benzene and substituted derivatives |  |
| 6.95_433.1502m/z | 139758-40-0^b^ | (4-Hydroxy-3,5-dimethoxyphenyl)[(3R,4R,5S)-tetrahydro-5-(4-hydroxy-3,5-dimeth... | C22H26O9 | -0.46 |  |  | Lignans, neolignans and related compounds | Furanoid lignans |  |
| 7.62_256.2646m/z | HMDB0012273 | Palmitic amide | C16H33NO | 4.42 | + | + | Lipids and lipid-like molecules | Fatty Acyls |  |
| 7.75_315.2780n | HMDB0038057 | Dehydrophytosphingosine | C18H37NO3 | 2.20 | + | + | Organic nitrogen compounds | Organonitrogen compounds | + |
| 7.79_293.1754m/z | HMDB0035722 | Tanacetol A | C17H26O4 | -1.62 | + | + | Lipids and lipid-like molecules | Prenol lipids |  |
| 8.02_302.3058m/z | HMDB0000269 | Sphinganine | C18H39NO2 | 1.51 | + | + | Organic nitrogen compounds | Organonitrogen compounds |  |
| 8.10_471.3478m/z | HMDB0033213 | (3alpha,20R,24Z)-3-Hydroxy-21-oxoeupha-8,24-dien-26-oic acid | C30H46O4 | 1.97 | + | + | Lipids and lipid-like molecules | Prenol lipids |  |
| 8.11_424.3354n | HMDB0006327 | alpha-Tocotrienol | C29H44O2 | 3.06 | + |  | Lipids and lipid-like molecules | Prenol lipids |  |
| 8.25_414.2037n | HMDB0039380 | Armillarin | C24H30O6 | -1.21 | + | + | Organic oxygen compounds | Organooxygen compounds |  |
| 8.43_678.3826n | HMDB0041094 | Gingerglycolipid B | C33H58O14 | -0.11 | + | + | Lipids and lipid-like molecules | Glycerolipids | + |
| 8.73_294.2198n | HMDB0301805 | 3-Oxo-2-(2-entenyl)cyclopentaneoctanoic acid | C18H30O3 | 1.01 | + | + | Lipids and lipid-like molecules | Fatty Acyls |  |
| 8.74_330.2407n | HMDB0004710 | 9,10,13-TriHOME | C18H34O5 | 0.27 | + | + | Lipids and lipid-like molecules | Fatty Acyls |  |
| 9.08_291.1960m/z | HMDB0030996 | (2'E,4'Z,7'Z,8E)-Colnelenic acid | C18H28O3 | -1.97 | + | + | Lipids and lipid-like molecules | Fatty Acyls |  |
| 9.08_485.3264m/z | HMDB0037963 | Actinidic acid | C30H46O5 | -1.79 | + |  | Lipids and lipid-like molecules | Prenol lipids |  |
| 9.36_634.3868n | HMDB0029780 | 2alpha-Hydroxypyracrenic acid | C39H54O7 | -0.20 | + | + | Lipids and lipid-like molecules | Prenol lipids |  |
| 9.67_442.2929n | HMDB0038242 | Ascorbyl stearate | C24H42O7 | -0.40 | + |  | Lipids and lipid-like molecules | Fatty Acyls |  |
| 9.82_290.1729n | HMDB0032959 | 1-Octen-3-yl glucoside | C14H26O6 | 0.02 | + | + | Lipids and lipid-like molecules | Fatty Acyls |  |
| 9.84_315.2538m/z | HMDB0002259 | Heptadecanoic acid | C17H34O2 | -0.88 | + | + | Lipids and lipid-like molecules | Fatty Acyls |  |
| 9.86_386.2669n | HMDB0036015 | Mangalkanyl glucoside | C21H38O6 | 0.18 | + | + | Organic oxygen compounds | Organooxygen compounds |  |
| 9.94_299.2592m/z | HMDB0037396 | xi-10-Hydroxyoctadecanoic acid | C18H36O3 | 0.11 | + | + | Lipids and lipid-like molecules | Fatty Acyls |  |
| 10.27_853.4609m/z | HMDB0041028 | Hovenidulcioside A1 | C44H68O16 | 3.38 | + | + | Lipids and lipid-like molecules | Prenol lipids |  |
| 10.37_325.2380m/z | HMDB0000673 | Linoleic acid | C18H32O2 | -1.47 | + | + | Lipids and lipid-like molecules | Fatty Acyls |  |
| 10.53_356.2934n | HMDB0034031 | 3-(2-Heptenyloxy)-2-hydroxypropyl undecanoate | C21H40O4 | 2.17 | + | + | Lipids and lipid-like molecules | Glycerolipids |  |
| 10.60_271.2275m/z | HMDB0006294 | 16-Hydroxyhexadecanoic acid | C16H32O3 | -1.33 | + | + | Lipids and lipid-like molecules | Fatty Acyls | + |
| 10.74_828.4898n | HMDB0013476 or HMDB0013517 or HMDB0013532 | PGP(16:0/18:1(9Z)) or PGP(18:1(11Z)/16:0) or PGP(18:1(9Z)/16:0) | C40H78O13P2 | -2.43 | + | + | Lipids and lipid-like molecules | Glycerophospholipids |  |
| 10.77_379.2858m/z | HMDB0036568 | Persenone A | C23H38O4 | 3.91 | + | + | Lipids and lipid-like molecules | Fatty Acyls |  |
| 10.92_884.4766n | HMDB0034053 | Polypodoside A | C45H72O17 | -0.42 | + | + | Lipids and lipid-like molecules | Steroids and steroid derivatives |  |
| 10.93_463.3059m/z | HMDB0035973 | Lucidenic acid M | C27H42O6 | 0.96 | + | + | Lipids and lipid-like molecules | Prenol lipids |  |
| 10.94_282.2572n | HMDB0000207 | Oleic acid | C18H34O2 | 4.69 | + | + | Lipids and lipid-like molecules | Fatty Acyls |  |
| 10.96_455.3515m/z | HMDB0002364 | Oleanolic acid | C30H48O3 | -3.43 | + |  | Lipids and lipid-like molecules | Prenol lipids |  |
| 11.03_901.4790m/z | HMDB0035481 | Tuberoside A (Allium tuberosum) | C45H74O18 | -1.40 | + | + | Lipids and lipid-like molecules | Steroids and steroid derivatives |  |
| 11.61_369.3003m/z | HMDB0242127 | Docosanedioic acid | C22H42O4 | -1.85 | + | + | Lipids and lipid-like molecules | Fatty Acyls |  |
| 11.61_381.3013m/z | HMDB0032735 or HMDB0041103 | Isopersin or Persin | C23H40O4 | 3.49 | + | + | Lipids and lipid-like molecules | Fatty Acyls |  |
| 11.66_278.0062n | HMDB0302296 | Arctic acid C | C13H10O3S2 | -3.35 | + | + | Organoheterocyclic compounds | Bi- and oligothiophenes |  |
