# Supplementary figures and images for: Combined metabolomics and bioactivity assays kernelby-productsof two native Chinese cherry species: The sources of bioactive nutraceutical compounds
Source: Food Chem X. 2024 Jul 5;23:101625. doi: 10.1016/j.fochx.2024.101625 (PMC11296007; doi:10.1016/j.fochx.2024.101625)

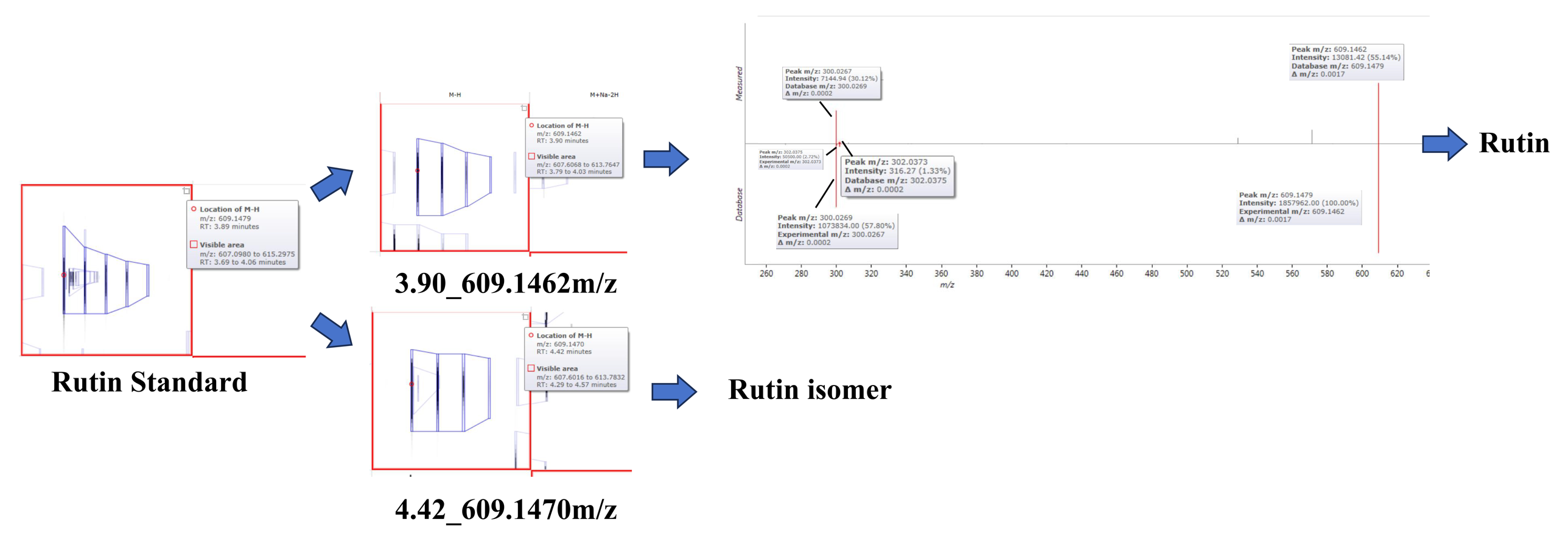

Supplement: Supplementary file 3 — Supplementary material 3 Compound 3.90_609.1462m/z was identified as Rutin by rutin standard and 4.42_609.1470m/z was identified as Rutin isomer. [file mmc3.zip › mmc3.jpg]
